# Lithology as a factor for the distribution of metals in stream sediments associated with sediment-hosted Cu deposits: a case study from the Alta-Kvænangen tectonic window, northern Norway

**DOI:** 10.1007/s10653-025-02387-y

**Published:** 2025-03-04

**Authors:** Laura Posarić, Sabina Strmić Palinkaš, Johan Hilmo, Željka Fiket, Andrea Čobić, Hana Fajković

**Affiliations:** 1 Department of Geology, University of Zagreb Faculty of Science, Horvatovac 102B, 10000 Zagreb, Croatia; 2 Department of Geosciences, UiT–The Arctic University of Norway, Dramsvegen 201, 9037 Tromsø, Norway; 3 Department of Earth Science, University of Bergen, Allégaten 41, 5007 Bergen, Norway; 4 Division for Marine and Environmental Research, Ruđer Bošković Institute, Bijenička Cesta 54, 10000 Zagreb, Croatia

**Keywords:** Stream and river sediments, X-ray powder diffraction (XRPD), Seven-step sequential extraction analysis, Spatial analysis, Principal component analysis (PCA), Sediment-hosted Cu deposits

## Abstract

The Kåfjord area in northern Norway hosts numerous Cu deposits that were subjected to mining activities back in the nineteenth century. Relicts of the historical mining activity are still visible at several abandoned mines and associated mine waste disposal sites that may represent an environmental threat. The area was subjected to mining activities during the nineteenth century and abandoned mines and associated mine waste disposal sites still may represent a significant environmental threat. The Cu mineralization, found within the Paleoproterozoic Alta-Kvænangen Tectonic Window, primarily occurs as epigenetic sulfide-quartz-carbonate hydrothermal veins that crosscut the Kvenvik volcano-sedimentary complex and the overlying Storviknes sedimentary sequence. This study aims to determine the geochemical composition of stream sediments associated with the sediment-hosted Cu deposits and examine the role of host lithologies in the dispersion of elements associated with the deposits. Sediments from two streams and a river in the Kåfjord area were analyzed using phase and element analyses (*aqua regia* chemistry), complemented by a seven-step sequential extraction procedure. Results from Annaselva stream, draining Cu occurrences in the carbonate sediments of the Storviknes sequence, showed a significant positive correlation of Cu with mobile chalcophile elements (Pb, Zn, Ni, Tl, Hg, Ag, Sb, Bi) and lithophile elements (Sr, Ca, Ba, Al, K). In contrast, Brakkelva stream, draining the mafic volcanics of the Kvenvik complex, exhibited no statistically significant correlations between Cu and any of the analyzed elements. Møllneselva River, draining both lithologies, showed a strong Cu-Sc correlation, with principal component analysis indicating limited distinction between lithology-derived elements. These results did not completely align with statistical analysis outcomes highlighting the challenges of statistical data interpretation using a limited number of samples.

**Supplementary Information:**

The online version contains supplementary material available at 10.1007/s10653-025-02387-y.

## Introduction

Stream and river sediments are often used in geochemical surveys, serving both as a tool for identifying perspective mining sites and as proxies indicating the environmental impact of mineral deposits and associated mining activities (Fletcher, 1997; Alexakis, 2011; Chu & Rediske, 2012; Zheng et al., 2014; Gus Djibril et al., 2016; Kirkwood et al., 2016; Salomão et al., 2021). In both cases, the geochemical haloes depend on numerous factors, including mineral assemblages of the primary mineralization, the trace element composition of individual mineral phases, the buffering potential of host rocks, and the hydrological dynamics in the catchment basin (e.g. Rose et al., 1979; Antunes et al., 2014; Langman et al., 2015; Grunsky & Caritat, 2020).

Copper (Cu) is one of the most important metals in the green energy transition, primarily due to its superior performance in electrical applications. Its importance is recognized by the European Commission, which labelled Cu as Critical Raw Material in 2023 (EC, 2023). Although Cu represents one of the essential micronutrients involved in a wide range of metabolic processes, at elevated levels it becomes toxic to both plants and animals (e.g. Flemming & Trevors, 1989; Gaetke & Chow, 2003; José Rodrigues Cruz et al., 2022) and therefore mining and smelting of Cu ores often represent an environmental threat. In addition to Cu, copper ore and its host lithologies can represent a source of various toxic metals, such as As, Cd, Hg, and Pb (Yin et al., 2012; Izydorczyk et al., 2021; Mun et al., 2021). Consequently, any interpretation of stream sediment analyses—whether in mineral exploration surveys or environmental studies—should consider the genetic aspects of Cu mineralization and related assemblages, such as residues from exploitation.

**Fig. 1:**
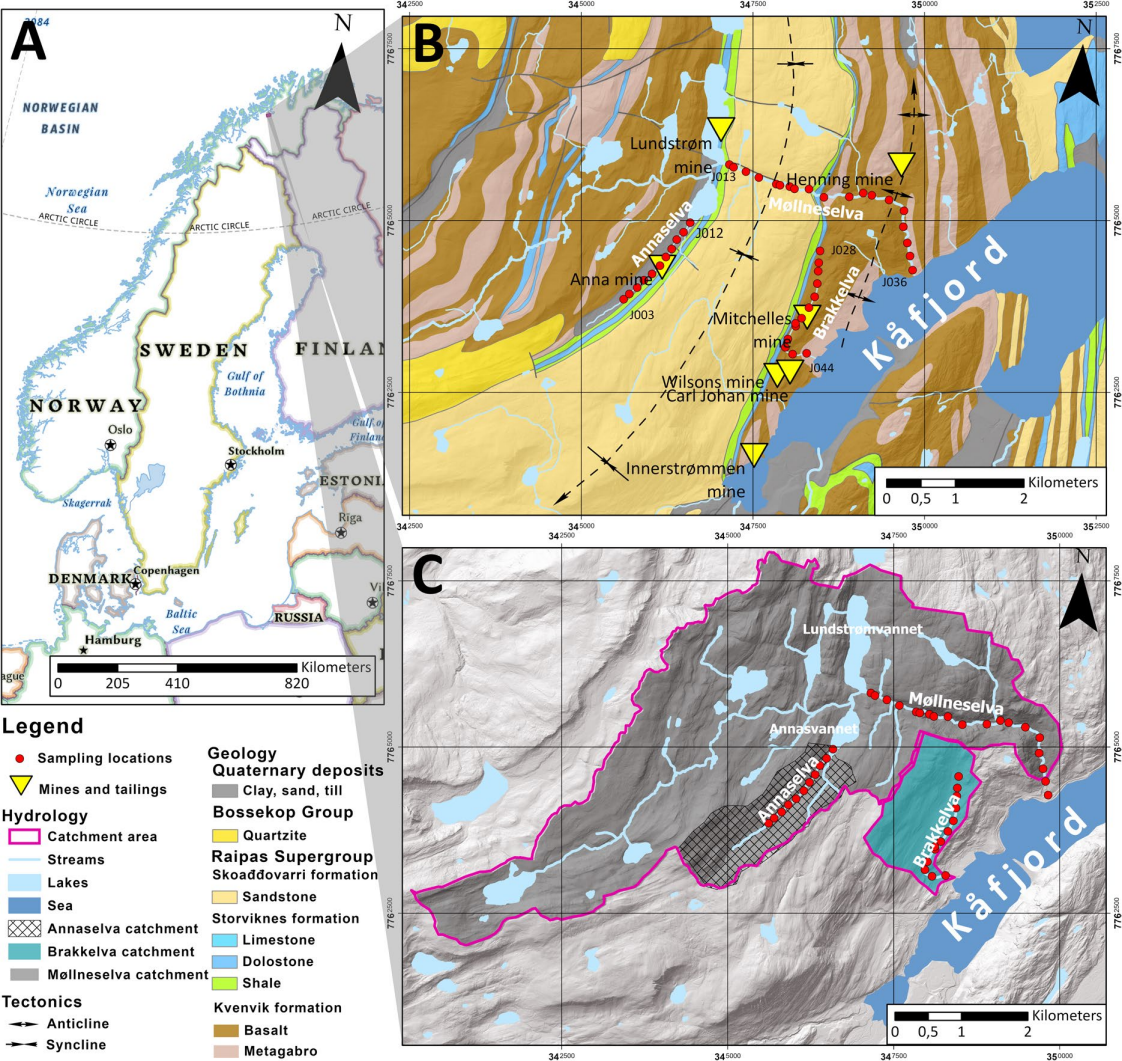
Geographical (**a**), geological (**b**), and hydrological (**c**) setting of the research area. The base map for the geographical setting map (a) was National Geographic Basemap from ArcGIS software (ESRI, 2022). Data for the geological and hydrological map – bedrock map were modified from the Geological Survey of Norway (NGU, 2022), bedrock map was modified according to Bergh and Torske (1988). The hydrological data were taken from the Norvegian Water Resources and Energy Directorate data services (NVE, 2022a, 2022b, 2022c). Sampling locations are marked with red dots and can be seen both on the geological map (only the first and last for each stream are labelled) and the hydrological map

For purposes of this research, the Kåfjord area of the Alta-Kvænangen Tectonic Window (AKTW) in northern Norway was investigated (Fig. 1). The study area has been selected because it exposes historically mined sediment-hosted Cu deposits and associated mine waste disposal sites (Vik, 1985; Eilu, 2012; Hilmo, 2021). Globally, sediment-hosted Cu deposits are responsible for approximately 20 percent of Cu production and, after porphyry Cu deposits, represent the most important source of Cu (Hayes et al., 2015). Therefore, the Kåfjord area represents an ideal natural laboratory to identify the geochemical footprint of this type of Cu mineralization.

The Kåfjord area is drained by several rivers and streams and in this study we focused on the Møllneselva River and the Annaselva and Brakkelva streams (Fig. 1), i.e. on three watercourses that traverse the Kvenvik volcano-sedimentary complex and the Storviknes sedimentary sequence, which are two main host lithologies for the Cu mineralization in the AKTW. The study area is covered by snow most of the year and weathering processes are strongly influenced by snow melting in the warmer months (Fletcher, 1997; Lana-Renault et al., 2011).

This study presents a suite of mineralogical and geochemical data obtained from the stream sediments, including the results of a 7-step sequential extraction analysis developed by Torres and Auleda (2013). A statistical approach has been employed to discern multi-element dispersion patterns associated with weathering of sediment-hosted Cu mineralization in the Alta-Kvænangen Tectonic Window. Particular attention is given to the distribution and binding sites of potentially toxic elements and whether the specific geological setup of AKTW is reflected in the geochemical analyses data. Additionally, a comparison of the statistical analysis with the sequential extraction results is provided, giving an insight into advantages and disadvantages of the chosen methods.

## Study area

The study area is located in the northern part of Norway, in Troms and Finnmark County (Fig. 1A). It lies above the Arctic Circle and it is characterized by subarctic climate, featuring very cold winters and short summers. Over the period from 1965 to 2020, temperatures in the area ranged from − 30 °C up to 32.5 °C, with an average annual temperature of − 1.3 °C. During the same period, the annual precipitation varied between 257 and 656 mm (NKSS, 2022).

The area represents a part of the Paleoproterozoic Alta Kvænangen Tectonic Window (AKTW, Fig. 1B), one of several exposures of the Precambrian Fennoscandian Shield within the Scandinavian Caledonides (Melezhik et al., 2015; Torgersen et al., 2016; Mun et al., 2020; Nasuti & Roberts, 2023). The AKTW is comprised of the sedimentary and volcanic rocks that are part of the Raipas Supergroup (Bergh & Torske, 1986; Fareth, 1979; Gautier et al., 1979; Melezhik et al., 2015). The supergroup is divided into several formations: the Kvenvik Formation, Storviknes Formation, Skoađđovárri Formation, and Luovosvárri Formation (Bergh & Torske, 1986; Melezhik et al., 2015). According to Melezhik et al. (2015) 2146 ± 5 Ma (U–Pb, zircon) obtained from a gabbro comagmatic with mafic lavas provides a minimum age for the deposition of the 13C-rich, Lower and Upper dolostones and the accumulation age of the 13C-rich Uppermost dolostone. The lower portion of the Kvenvik Formation, a volcano-sedimentary complex, is composed mostly of gabbro intercalated with layers of dolostone, albite felsites, shale, albite-carbonate-magnetite rocks, mafic tuff, and tuffite. The entire area has been subjected to greenschist facies metamorphism. The upper portion of the Kvenvik formation consists of mafic tuffs, massive and pillow tholeiitic basalt, intercalated with layers and lenses of dolostone, limestone, and black shale. The Storviknes Formation is primarily composed of dolostones with stromatolites, dolostone breccias, and purple and grey siltstone (Melezhik et al., 2015). The Skoađđovárri formation is predominantly composed of sandstone interbedded with conglomerate, pebbly sandstone, and shale. The Luovosvárri Formation is made up of dolostone and sandstone (Gautier et al., 1979; Melezhik et al., 2015). The details on geological features in the study area, including the geological column, can be found in Melezhik et al. (2015). The Raipas Supergroup has been subjected to greenschist facies metamorphism (Melezhik et al., 2015).

The AKTW hosts numerous sedimentary-hosted Cu deposits. The mineralization in AKTW predominantly occurs in the form of sulfide-quartz-carbonate hydrothermal veins that crosscut lithologies of the Kvenvik volcano-sedimentary complex and the overlying Storviknes sedimentary sequence (Eilu, 2012; Melezhik et al., 2015; Simonsen, 2021). Even in the Kvenvik formation, the Cu mineralization is predominantly hosted by layers and lenses of carbonates in the volcano-sedimentary complex. Only locally epigenetic quartz-carbonate-sulfide veins that crosscut both gabbroic and tuffitic rocks can be found. Anyway, these features are common in so-called low-grade zone of sediment-hosted Cu deposits. Furthermore, the genetic model suggest that mafic rocks in the area were source of Cu (Simonsen, 2021) similar to some other sediment-hosted Cu deposits elsewhere (Sanislav et al., 2023). Pyrite and chalcopyrite are prevailing sulfide minerals found in the veins hosted by the Kvenvik lithologies. In sediment-hosted Cu deposits, the low-grade zone is characterized by relatively low Cu activity and pyrite is still a stable sulfide phase. As the Cu activity increases, Cu-sulfides such as chalcopyrite, bornite, digenite, replace pyrite (Hitzman et al., 2010). In contrast, the sulfide mineralization in the Storviknes sedimentary sequence is more complex and consists of chalcopyrite, bornite, digenite, galena, covellite, wulfenite, tennantite, molybdenite, and wittichenite (Simonsen, 2021). The mineralization that crosscut the Storviknes sediments still has the sulfide-quartz-carbonate load characteristics, but in contrast to the mineralization in the Kvenvik formation that has a low-grade character, here the high-grade mineralization with more diverse Cu-sulfide mineralogy is present. Again, this is common for the sedimenet-hosted Cu deposits globally (Hitzman et al., 2005, 2010). The mining of Cu in the Kåfjord area started in 1827 and lasted until 1878, during which more than 62,000 t of cobbed ore was produced. Mining continued after 1895 and lasted until 1908, in which period 5000–6000 t of Cu ore were extracted (Eilu, 2012).

**Fig. 2:**
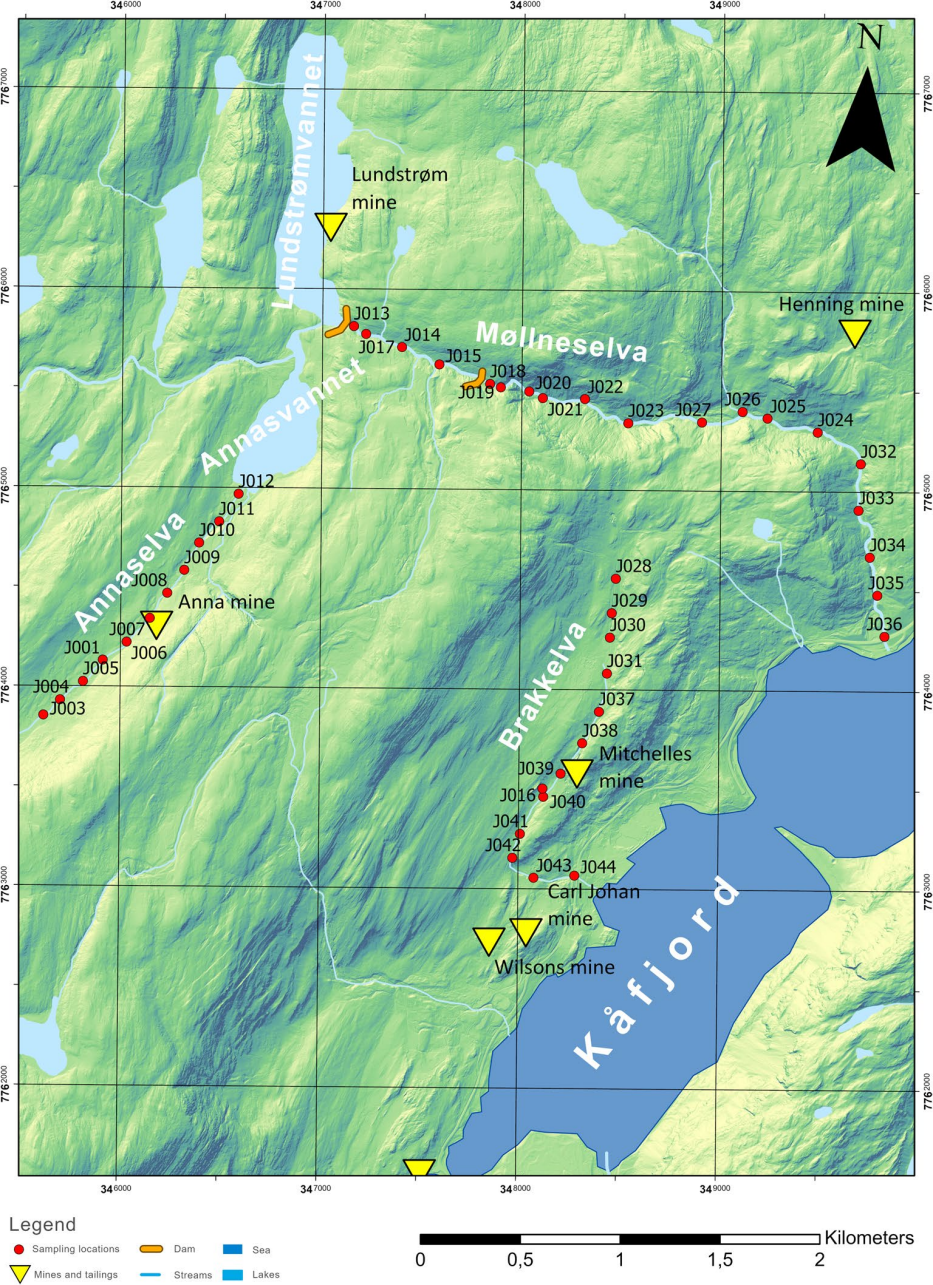
Map with the sampling locations and locations of the historical mines and tailings. Dam locations are approximate and can differ slightly in the field, but the relationships between samples and dams are kept, as indicated in Hilmo (2021)

The hydrological setting of the study area involves the Møllneselva River and two streams: Annaselva and Brakkelva (Fig. 1C). Brakkelva drains the Cu deposits hosted by the Kvenvik volcano-sedimentary complex (Fig. 1). Annaselva and its catchment are tributary to the larger catchment of the Møllneselva River. Annaselva drains the Cu deposits hosted by carbonate rocks of the Storviknes sedimentary complex, while the Møllneselva River crosscuts both the Kvenvik and Storviknes formations and runs over several different lithologies including sandstones, metabasalts, metagabbro, metadolostone, limestone and shales (Fig. 1). The Møllneselva River is divided by two dams. The first dam is located at the SE end of Lake Lundstrømvannet, and after the dam river Møllneselva continues its course. The second dam is in the middle of the Møllneselva River upstream from sample J018. The positions of dams can be found in Fig. 2. The Brakkelva catchment covers an area of 2.2 km^2^, with the lowest elevation at 8 m above mean sea level (AMSL) and the highest elevation at 569 m AMSL (Fig. 1C). The area of the Annaselva catchment is 2.45 km^2^, with a minimum elevation of 480 m AMSL and maximum elevation of 901 m AMSL (Fig. 1C). The Møllneselva catchment is the largest of the three with an area of 22.87 km^2^. Its highest elevation is at 1110 m AMSL, while the lowest is at 0 m AMSL (Fig. 1C).

## Materials and methods

### Samples and sample preparation

The sampling was conducted in the Alta region, NW of Kåfjord, and covered two streams Annaselva and Brakkelva, and the Møllneselva River (Fig. 1A). In total, sediments were sampled at 43 locations, with distances between locations ranging from 150 to 250 m. In total, 11 samples were taken from the Annaselva streambed, 19 samples from the Møllneselva riverbed, and 13 from the Brakkelva streambed. The sampling locations are listed in Online Resource 1 and presented in Fig. 2.

Sampling of the stream sediments required the removal of the uppermost 1–2 cm layer to minimize variations in a material due to stream flow. Sampling was conducted using a plastic soil trowel to scoop sediments into zip-lock plastic bags. All collected samples represent sediment samples.

After collection, samples were freeze-dried prior to sieving. Freeze drying was performed using a CHRIST ALPHA 1–4 LSC Plus freeze dryer. Dry sieving was conducted using sieves with mesh sizes of 0.063 mm, 0.125 mm, 0.250 mm and 1 mm. Each sample was sieved for 12 to 15 min using a Retsch AS 200 basic vibrator sieve shaker with an amplitude of 70%. After dry sieving, samples were collected in glass bowls and left to completely dry at 40 °C. The dried samples were stored in plastic zip-lock bags. The fraction < 0.063 mm was used in this study. An overview of the specific analyses carried out on the samples is given in Online Resource 1.

### Mineral composition analysis

The mineral composition analysis was conducted on 43 stream sediment samples of the < 0.063 mm fraction (Online Resource 1). A qualitative X-ray powder diffraction analysis (XRPD) was applied. Prior to the analysis, samples were manually ground in an agate mortar. The mineral composition was determined using a Philips PW 3040/60 X’Pert PRO powder diffractometer (Panalitycal B.V., Eindhoven, Netherlands) with CuKα radiation from the tube at 40 kV and 40 mA, collecting X-ray diffraction data from 4 to 65° 2Θ.

Five selected samples (Online Resource 1) were analysed for their clay mineral composition using qualitative XRPD analysis, using oriented mounts as described by Starkey et al. (1984). Samples were chosen based on their geographical position at the beginning and end of Annaselva and Brakkelva streams, and only at the end of Møllneselva. Only one sample from Møllneselva was taken because Annaselva flows into Møllneselva just before the first sample taken in the Møllneselva riverbed.

Prior to the clay mineral analysis, the carbonate fraction was removed from each sample using 1:10 hydrochloric acid (HCl) solution. The reaction was allowed to run for four days with occasional stirring. In the continuation of the analysis the organic fraction was removed using 1:1 hydrogen peroxide (H_2_O_2_). Subsequently, the samples were washed with distilled water, stirred, placed in plastic cuvettes, and centrifuged to separate the < 0.002 mm fraction (for details, see Starkey et al., 1984). The suspension containing this fraction was dripped on the glass slides and left to air dry. Once dried, the samples were analyzed using a Philips PW 3040/60X’Pert PRO powder diffractometer (Panalitycal B.V., Eindhoven, Netherlands) with CuKα radiation from the tube at 40 kV and 40 mA, collecting X-ray diffraction data from 4° to 65° 2Θ in the beginning of the analysis, and 4° to 30° 2Θ after each subsequent treatment. The subsequent steps involved treating oriented mounts with ethylene glycol, and heating the samples at 400 °C and 550 °C over periods of 4 h.

### Geochemical analysis of samples

The chemical composition of the samples was determined on a fraction < 0.063 mm in Bureau Veritas Mineral Laboratories, Vancouver, Canada. The fraction was treated by *aqua regia* digestion. After digestion, extracts were analysed by ultratrace ICP-MS method. Concentrations of 36 elements were analysed (Ag, Al, As, Au, B, Ba, Bi, Ca, Cd, Co, Cr, Cu, Fe, Ga, Hg, K, La, Mg, Mn, Mo, Na, Ni, Pb, S, Sb, Sc, Se, Sr, Te, Th, Ti, Tl, U, V, W, Zn). The analyzed batch of samples comprised 43 samples, with 7 samples being replicated. The data trends were visualized using the “ggplot2” package in RStudio (Wickham, 2016; RStudio Team, 2022).

### Sequential extraction procedure

#### Preparation of extracts

Sequential extraction (SE) is an analytical method commonly used to identify metals bound onto different solid phases in soils and sediments. There are numerous sequential extraction protocols, like frequently used the European Community Bureau of Reference (BCR) method (Rauret et al., 1999; Pueyo et al., 2008), the five-step sequential extraction according to Tessier (Tessier et al., 1979), and many more (Filgueiras et al., 2002; Rao et al., 2008). For the purposes of this research, a slightly modified seven-step SE analysis for sediments affected by acid mine drainage was conducted, as proposed by Torres and Auleda (2013).

According to these authors, the first step of the seven-step SE targets the water-soluble fraction. Mass of 0.5 g of the < 0.063 mm fraction was treated with oxygen-free deionized water. Oxygen-free deionized water was obtained by nitrogen purging, as described by Butler et al. (1994). The second step extracts an exchangeable fraction. The undissolved residual material from first step is treated with 1 mol/dm^3^ ammonium acetate. In the third step, poorly crystalline Fe(III)-oxyhydroxides are treated using 0.2 mol/dm^3^ ammonium oxalate in dark, while in the fourth, crystalline Fe(III)-oxides are targeted by 0.2 mol/dm^3^ ammonium acetate, again. In the fifth step, organic fraction is treated with sodium hydroxide (NaOH), while in the sixth step, sulfides are the targeted fraction and are treated with 8 mol/dm^3^ nitric acid (HNO_3_). The seventh step of the Torres and Auleda (2013) SE analysis targets residual fraction, i.e., primary and secondary silicate minerals, and resistant minerals such as rutile and zircon. The residual fraction is treated by combination of strong acids (HNO_3_, HCl, HF), which is explained in detail in the text below.

Sequential extraction was carried out on 14 samples (Online Resource 1). Samples were selected based on their geographical position: at the beginning and the end of the sampled stream, with an additional two – three samples in-between (Fig. 2). To assess the precision of the analysis, one sample was prepared in triplets. Additionally, a blank sample was also prepared.

After digestion with solvent according to instructions, samples were centrifuged, and solutions were filtered through FilterBio® PES Syringe filters, 25 mm in diameter, with pore sizes of 0.22 or 0.45 μm (depending on the instructions for each step). The extractions were performed in PET bottles and PET laboratory dishes that were washed in 10% HNO_3_. Additionally, a sand bath was used for heating the extracts. The temperature of the sand bath was set at 80 ± 5 °C.

The seventh step was modified. The residuals from step six were dried, and 0.05 g was taken for the last seventh step of the SE. Samples were treated with mixture of 4 ml nitric acid (HNO_3_, 65%, *pro analysi*, Kemika, Zagreb, Croatia), 1 ml hydrochloric acid (HCl, 36.5% *pro analysi*, Kemika, Zagreb, Croatia) and 1 ml hydrofluoric acid (HF, 48% *pro analysi*, Kemika, Zagreb, Croatia) followed by addition of 6 ml boric acid (H_3_BO_3_, Fluka, Steinheim, Switzerland). The total digestion in the seventh step was assisted using a microwave oven. The perchloric acid (HClO_4_) from the instructions by Torres and Auleda (2013) was substituted for HF. This procedure represents an established practice when using ICP-MS for analysis. Additional steps and digestion procedure are explained in detail by Fiket et al. (2016). The precision of the seventh step was checked by analysing two samples in duplicates and one in quadruplets.

### Multielement analysis by ICP-MS-QQQ

Multielement analysis of the prepared extracts by seven-step SE analysis was performed by Inductively Coupled Plasma Mass Spectrometry with an additional quadrupole mass filter (ICP-MS-QQQ) using an 8900 ICP-QQQ instrument (Agilent, Santa Clara, USA). All extracts were diluted 100-fold or tenfold (for the seventh step), acidified with HNO_3_ (68%, supra pur), and an internal standard was added (In; 1 μgl^−1^). Typical instrument conditions and measurement parameters used in this analysis are given in Online Resource 2, while a detailed workflow of the method can be found in Petrović et al. (2023).

All samples were analysed for the total concentration of 28 elements (Al, As, Ba, Be, Ca, Cd, Co, Cr, Cu, Fe, K, Li, Mg, Mn, Mo, Ni, Pb, Rb, Sb, Sc, Se, Sn, Sr, Ti, U, V, Y and Zn). The quality control of the analytical procedure was conducted by simultaneously analyzing the blank sample and the certified reference material for soil (NCS DC 77302, China National Analysis Center for Iron and Steel, Beijing, China).

### Statistical analysis

Statistical analysis was conducted using the RStudio computer software (RStudio Team, 2022), and all statistical data were visualized using the R packages “tidyverse” (Wickham et al., 2019) and “ggplot2” (Wickham, 2016).

For the statistical analysis, values below the lower limit of detection (LLD) and values above the upper limit of detection (ULD) were transformed in a semi-total geochemical analysis dataset. Values below LLD were established as the LLD value multiplied by 0.65, as Palarea-Albaladejo and Martín-Fernández (2013) proved that it works reasonably when the number of data below the detection limit is low. In the bulk geochemistry dataset, less than 5% of the values were below LLD for all elements included in the statistical analysis, except for Te, which had 20% of the values below the LLD. Conversely, in the same dataset, concentration values above ULD were transformed by multiplying the ULD values with 1.2, as demonstrated by Mikšová et al. (2020) to be effective when the percentage of data above ULD is low. In this case, that accounted for 2.3% of the values for Mn.

The number of LLD values in the SE dataset was higher than in bulk geochemistry dataset. However, as no advanced statistical analysis was carried out on the SE data (except for descriptive statistical analysis), the number of LLD values is not of critical importance.

Both datasets were analysed to obtain descriptive statistical values, including the minimum value, 1st quartile, mean, median, 3rd quartile, and maximum value. These values are presented in violin plots for semi-total geochemical analysis data using measured values, while sequential extraction values are depicted in box and whiskers plots after the *log10* transformation of the values for better visualization.

The bulk geochemistry dataset underwent further geostatistical analysis. The bulk geochemistry dataset is described as compositional data, for which careful preparation and treatment of the data is necessary (Jones and Aitchinson, 1982; Aitchison, 1992; Carranza, 2011; Mueller et al., 2020; Blannin et al., 2022a, 2022b; Blannin et al., 2023). The analysis included calculating variation of data based on pairwise log-ratios for all measured elements (Kynčlová et al., 2017). Variation was calculated on closed data (using “*acomp”* and *“variation”* commands in “compositions” package for RStudio, van den Boogaart & Tolosana-Delgado, 2013; van den Boogaart et al., 2022) for each stream and river separately. This helped create subsets that were analysed separately. The pairwise log ratios for each pair of elements were visualized using heatmaps. The heatmap shows the correlation of the chemical elements, with lower values of the ratios indicating stronger and more significant positive correlations.

The variation heatmaps (Kynčlová et al., 2017) were used to select chemical elements that either group based on the values of the log-ratios or are significant for the area of research. Chosen elements are representative elements that can explain the behaviour of other elements in the area. Copper was the only element initially chosen for each subset as the main ore element from the area. After selecting elements, a principal component analysis (PCA) was conducted.

PCA is a multivariate statistical method used for reduction of dimensionality of data without losing important information (Kassambara, 2017). It is frequently utilized in exploration and environmental geochemistry to reveal hidden relationships between various observed variables (Passos et al., 2010; Tokalıoğlu et al., 2010;  Celauro et al., 2014; Gus Djibril et al., 2016; Acosta-Góngora et al., 2022).

For this research a set of 9 elements were chosen (Al, K, Cu, Ag, Ca, Sr, Ni, Zn, and Sc) for PCA analysis. The elements were chosen based on their geochemical role in the study area i.e. these show bigger dispersion and spatial variation (for each a detailed explanation is given in results and discussion section). The PCA analysis was carried out using the “stats” R package. Before PCA analysis, data were closed and centred using *acomp* command from “compositions” R package (van den Boogaart et al., 2022), while the results were visualized using “factoextra” R package (Kassambara & Mundt, 2020).

### Spatial analysis

Spatial analysis of the semi-total geochemical analysis data was performed using ArcGIS PRO 2.5.0 software (Esri Inc, 2020). A digital elevation model (DEM) of terrain file was provided from the “Geonorge” website (Norwegian Mapping Authority, 2024), while lakes, streams, and rivers were obtained from Norwegian Water Resource and Energy Directorate (NVE) website (NVE, 2022b). The DEM served as the background for all maps (e.g. Figure 1 and 2) with different colour ramps applied. The sources of other shapefiles and raster data are noted in figure descriptions. The spatial analysis of semi-total geochemical analysis data visualized concentrations of nine elements (Ag, Bi, Cr, Cu, Hg, Mo, Ni, Pb, Sb) at their sampling sites in the main part of the text, while all other elements can be found in supplementary material.

## Results and discussion

### Mineral composition

**Fig. 3:**
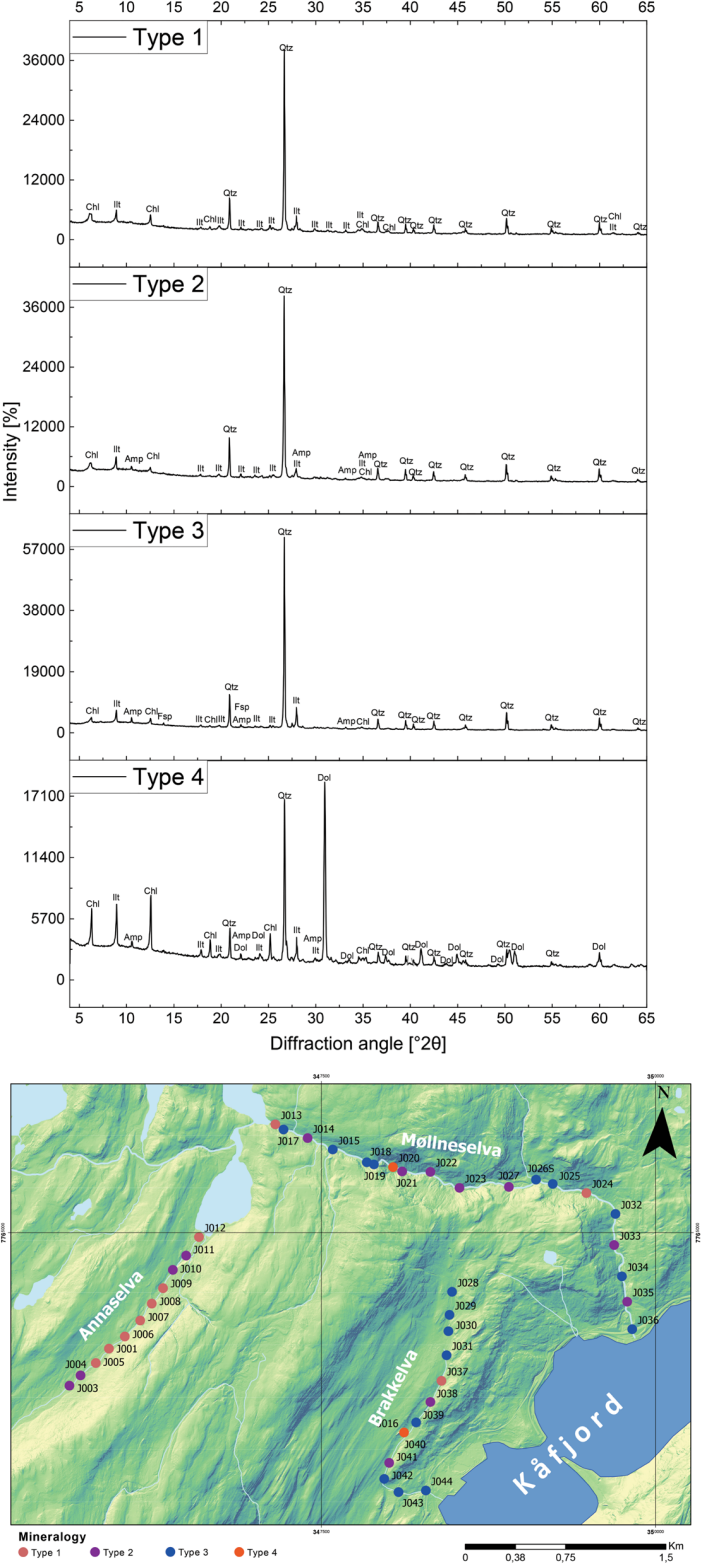
The XRPD patterns of representative samples (J013, J017, J038, J016) from 4 types of stream sediments from the study area and spatial visualization of samples according to the mineralogical types. Abbreviations: Qtz-quartz, Ilt – illite, Chl – chlorite, Amp – amphibole, Fsp – feldspar, Dol – dolomite (mineral abbreviations after Whitney & Evans, 2010)

**Fig. 4:**
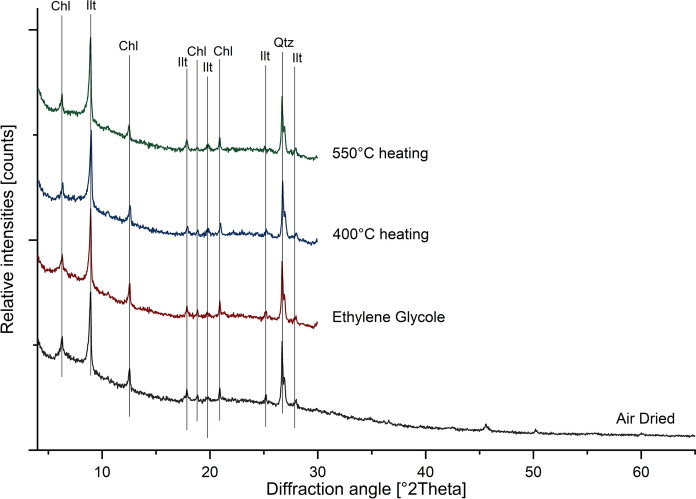
Identification of clay minerals in representative sample (shown J043). Abbreviations: Chl – chlorite, Ilt – illite, and Qtz – quartz (mineral abbreviations after Whitney & Evans, 2010)

Based on the bulk XRPD analysis, the samples can be subdivided into four main types (Fig. 3): 1) Type 1 contains only clay minerals and quartz; 2) Type 2, in addition to clay minerals and quartz, also shows the presence of amphiboles; 3) Type 3 contains clay minerals, quartz, amphiboles, and feldspars; and 4) Type 4 contains clay minerals, quartz, amphiboles, and dolomite. The clay mineral analysis suggests that chlorite and illite are prevailing clay phases in all four groups (Fig. 4).

Samples of all four types (Fig. 3) were identified in the stream sediments from Brakkelva and Møllneselva. In contrast, Annaselva’s stream sediments mostly belong to Type 1 (quartz and clay minerals), with occasional occurrences of Type 2 (Fig. 3, quartz, clay minerals and amphiboles).

As shown in the geological map (Fig. 1B), the obtained XRPD data reflect the geological setting of the Kåfjord area. The strong quartz maxima in all XRPD data types originate from quartzite and sandstone, as well as mineralized sulfide-quartz-carbonate veins. Abundant quartz makes identification of other mineral phases difficult. The absence of carbonate phases in Annaselva’s stream sediments is unexpected. Even though Fig. 1 shows Annaselva flowing over Quaternary deposits, surrounding area is composed of carbonates. It would be expected that in the area where physical weathering is the most important type of weathering (Millot et al., 2003), such as it is in the study area, carbonates surrounding the Annaselva stream would be transferred to the areas of lower altitude, i.e. the streambed itself, by surface runoff (Bačani, 2006). However, this is not the case in the study area and will be addressed further in the text.

### Chemical composition

**Fig. 5:**
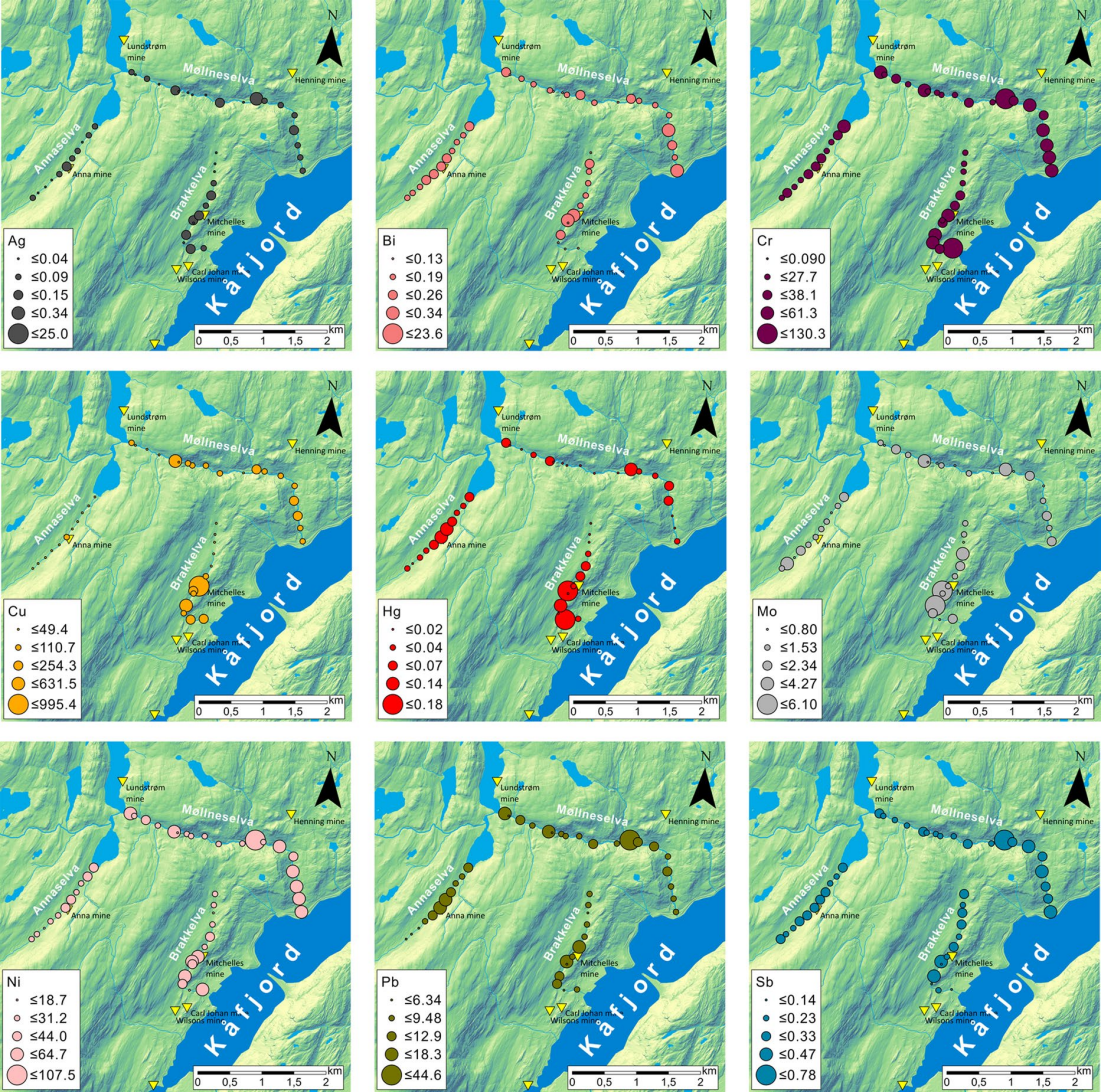
Spatial distribution of the semi-total geochemical concentration levels of metals of interest in the Kåfjord area. The size of the circles corresponds to the concentration, with larger circles representing higher concentrations

**Fig. 6:**
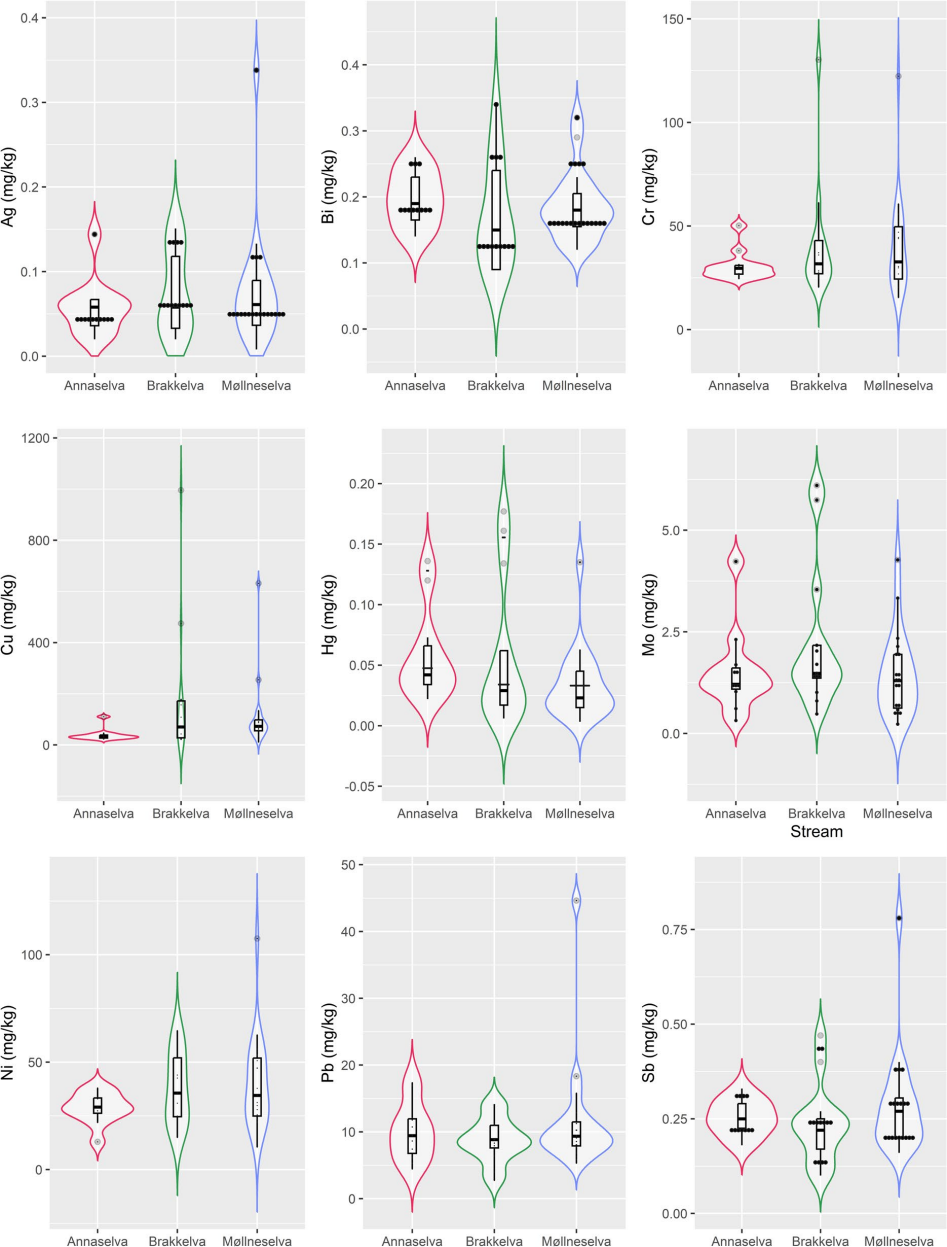
Violin diagrams with box diagrams displaying concentrations of elements of interest for stream sediments collected from Annaselva, Brakkelva, and Møllneselva. The diagrams depict minimum value, 1st quartile, median, mean, 3rd quartile, and maximum values in addition to the distribution of the data (curve of the violin diagram). A long “violin neck” is characteristic of the values that were measured, but have low probability of the occurrence. In other words, “bellies” in violin diagrams represent a higher frequency of occurrence of some value, while “narrow necks” show a low frequency of occurrence of the values, depicting the distribution of the data. The visualization was obtained with “ggplot2” (RStudio Team, 2022; Wickham, 2016)

Results of the semi-total geochemical analysis are listed in Online Resource 3. The spatial distribution of selected metals is shown in Fig. 5 and Online Resource 5, while concentration ranges are illustrated as violin and box plots (Fig. 6 and Online Resource 6). Nine elements (Ag, Bi, Cr, Cu, Hg, Mo, Ni, Pb, and Sb) are visualized, exhibiting higher concentrations near mine entrances and disposed tailing material (Figs. 5 and 6). These metals and their concentrations vary from stream to stream in the area, likely due to differences in lithology. For instance, Cr shows higher concentrations in Brakkelva and part of Møllneselva, i.e. areas where mafic rocks are present (Fig. 5), and there’s similar behaviour observed for Ni. These trends are observed for other elements in Online Resources 5 and 6, which indicate higher concentrations in Annaselva and Brakkelva (Ba, Sr, U, Zn) than in Møllneselva, or again higher concentrations in Brakkelva and Møllneselva (Cd, Co, Mg, etc.) than in Annaselva.

Semi-total geochemical analysis reveals an increase in concentrations of different elements (Fig. 5 and Online Resource 5), at points directly adjacent to the mine and tailings material (Fig. 2) where a direct mobilisation of metals occurs, the dam where the energy of water is lower (Fig. 2), leading to material accumulation and higher concentrations of metals due to lower flow velocity (Williams et al., 1973; Lu et al., 2022), as well as in samples J026 and J016 which were sampled at the inlets of two creeks which again have lower flow velocity and water capacity (Figs. 2 and 5 and Online Resource 5). In general, the highest concentrations for all measured metals are observed in Brakkelva and Møllneselva than in Annaselva (Fig. 6, and Online Resource 3 and 6).

**Fig. 7:**
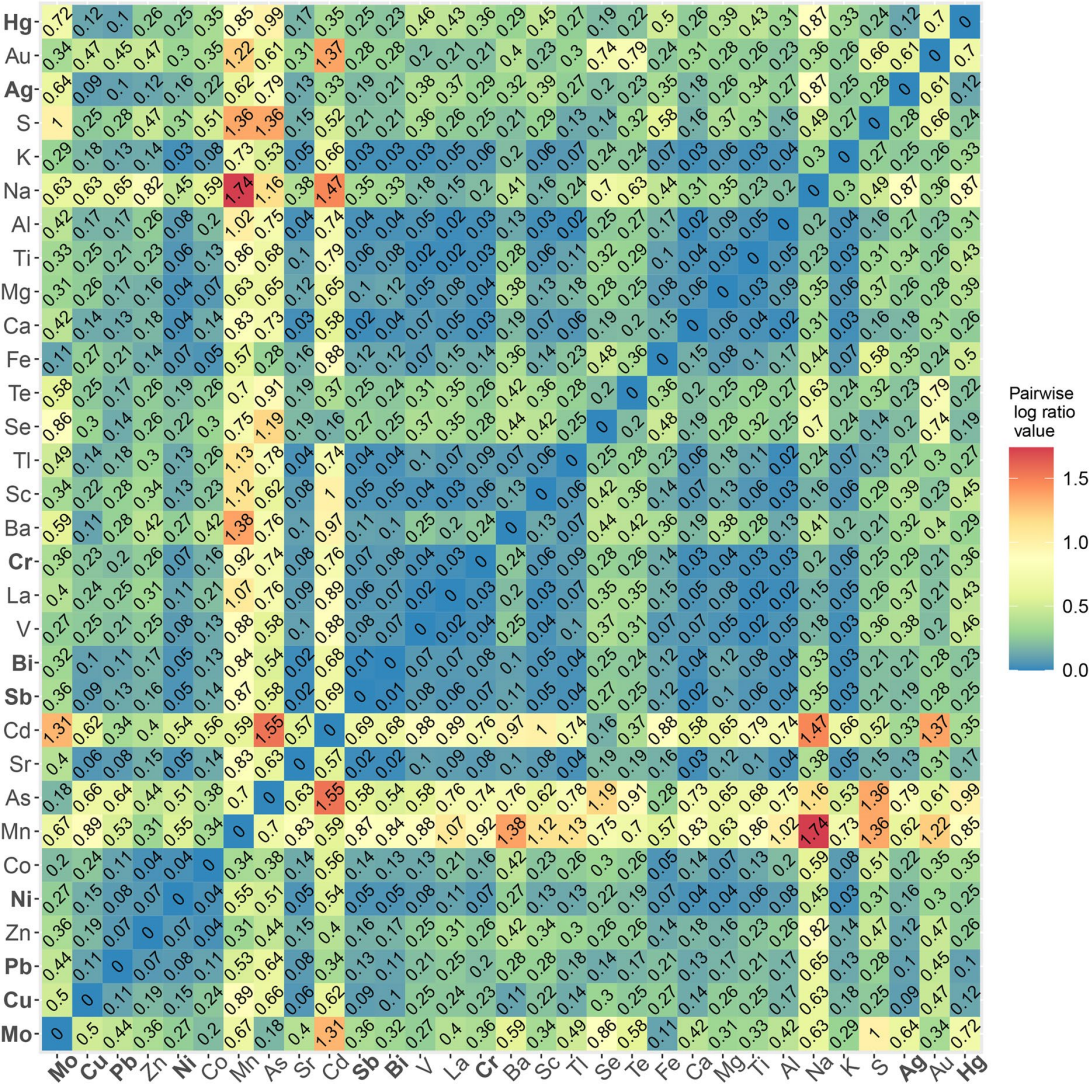
Variation of the data based on pairwise *log*-*ratios* of the bulk geochemistry. Data obtained from the Annaselva stream. Elements in bold are visualized in Figs. 5 and 6

Copper in the Annaselva stream sediments shows a significant positive correlation (*log-ratio (r)* ≤ *0.1*) with a range of chalcophile elements (Fig. 7): Ag (*r* = *0.09*), Sb (*r* = *0.09*), Bi (*r* = *0.10*); and some lithophile elements (Sr, *r* = *0.06*). Considering positive correlations where the *log-ratio* is below 0.2, additional chalcophile elements include Pb (*r* = 0.11), Zn (*r* = 0.19), Ni (*r* = 0.15), Tl (*r* = 0.14), Hg (*r* = 0.12), and lithophile elements Ba (*r* = 0.11), Ca (*r* = 0.14), Al (*r* = 0.17), K (*r* = 0.18). Data analysis reveals a strong connection between Cu and lithophile elements like Sr and Ca in the Annaselva stream. The carbonates are the main source for Ca and Sr in the Annaselva, as demonstrated by Hilmo (2021). The *log-ratios* of semi total geochemical analysis data therefore highlight correlations not evident in XRPD data.

**Fig. 8:**
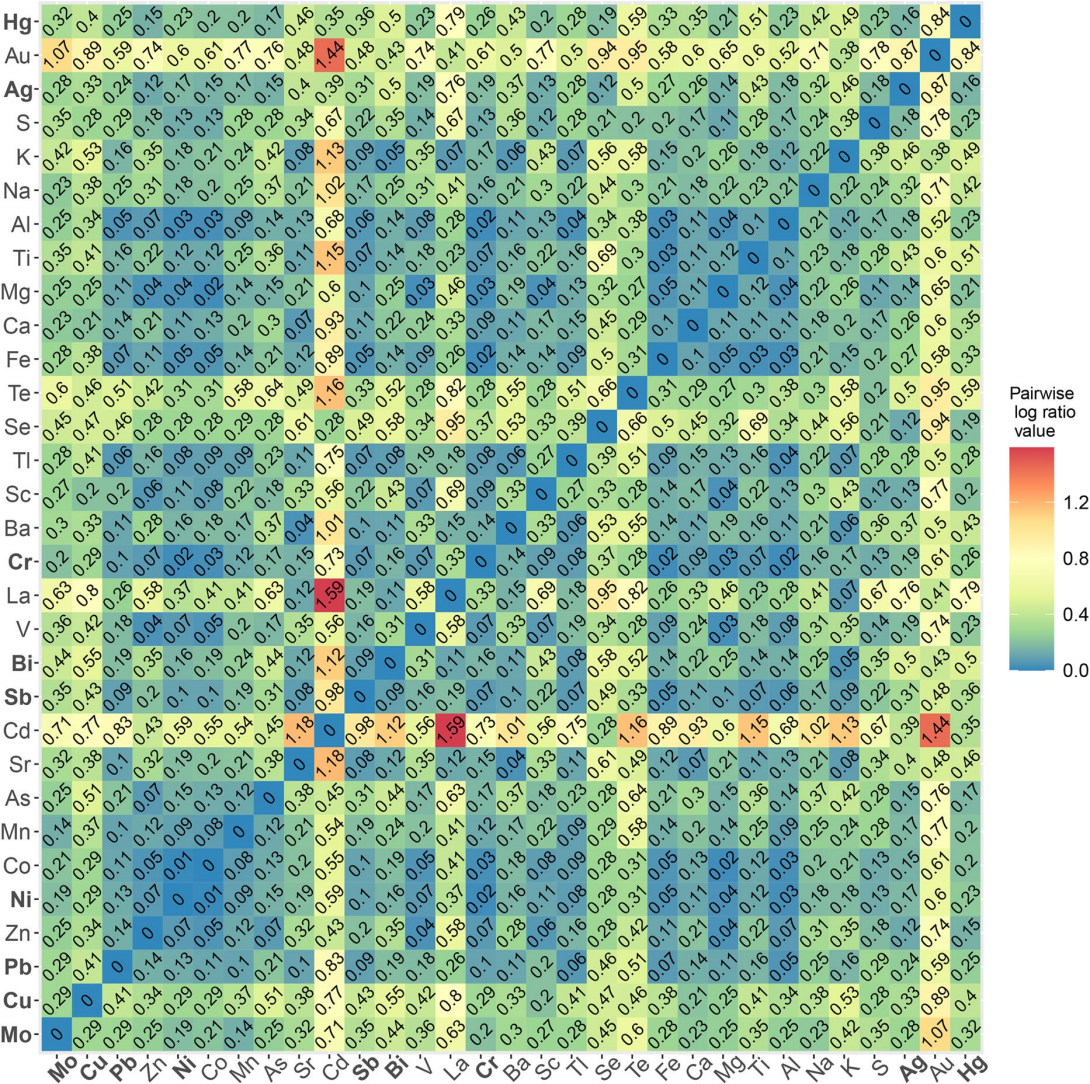
Variation of the data based on pairwise *log*-*ratios* of the bulk geochemistry. Data obtained from the Møllneselva River. Elements in bold are visualized in Figs. 5 and 6

For the Møllneselva and Brakkelva, the elements with *log-ratios* lower than 0.2 were taken into consideration as well. Møllneselva drains both the Storviknes sedimentary sequence and the Kvenvik volcano-sedimentary complex (Fig. 1). Copper in the Møllneselva River sediments does not show strong positive correlation with any of the analysed elements (Fig. 8), except for Sc. The Cu *log-ratio* value to Sc is *r* = 0.2.

**Fig. 9:**
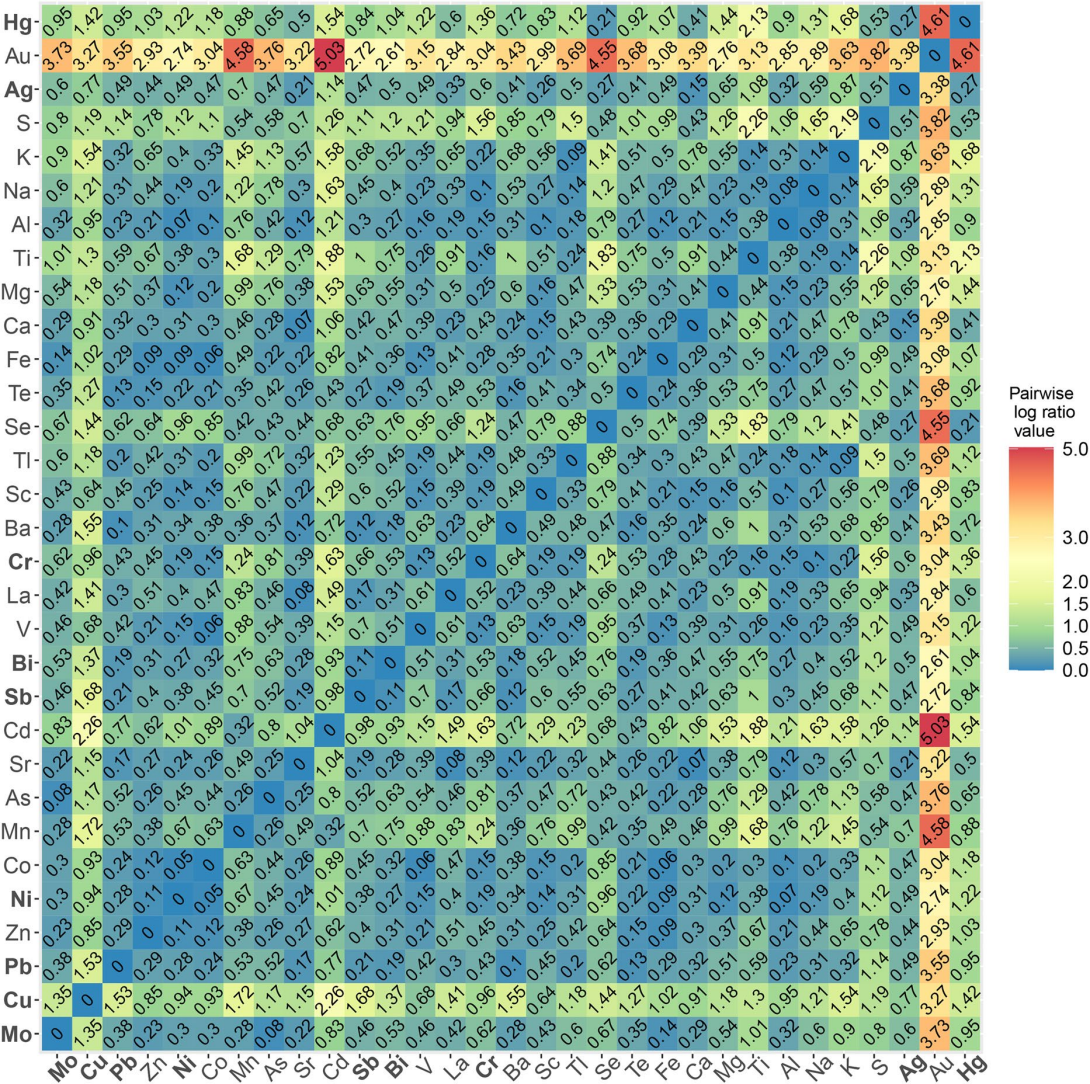
Variation of the data based on pairwise *log*-*ratios* of the bulk geochemistry data obtained from the Brakkelva stream. Elements in bold are visualized in Figs. 5 and 6

Brakkelva drains the Kvenvik volcano-sedimentary complex (Fig. 1). Copper in the sediments from this stream does not show strong positive correlations with the analysed elements (Fig. 9). The lowest *log-ratio* for Cu is with Sc (*r* = 0.64), a correlation also observed with stronger coefficient in Møllneselva River. Unlike in Annaselva, where lithological influence is evident in the semi-total geochemical analysis, such observations are absent in Møllneselva and Brakkelva.

Since deposits in the study area are Cu-sulfide, the correlations between sulfur (S) and other elements can provide insights into the redox potential (Eh) of the area. The lack of significant correlation between S and chalcophile elements in Brakkelva stream sediments suggests more intensive oxidation of sulfide minerals (Sato, 1960). In Annaselva stream sediments, S positively correlates with Tl (*r* = 0.13) and Se (*r* = 0.14). In Møllneselva River sediments, S shows significant positive correlations with various elements, including Ag (*r* = 0.18), Al (*r* = 0.17), Ca (*r* = 0.17), Co (*r* = 0.13), Cr (*r* = 0.13), Fe (*r* = 0.20), Mg (*r* = 0.11), Ni (*r* = 0.13), Sc (*r* = *0.12*), V (*r* = 0.14) and Zn (*r* = *0.18*). This relationship might be due to the higher concentrations of these metals in Møllneselva, especially in the lower part of the catchment. The positive correlations of S with chalcophile elements (Ag, Se, Tl, and Zn) in Møllneselva and Annaselva correspond to the detection of these elements in the pyrites and chalcopyrites collected in the area and analysed by LA-ICP-MS (Hilmo, 2021). This positive correlation of S and aforementioned chalcophile elements in sediments corresponds to the presence of sulfides in stream sediments determined by Hilmo (2021). Additionally, the correlation between S and Ca in Annaselva and Møllneselva, as well as S and Sr in Annaselva, supports the explanation of coupled sulfide oxidation and carbonate dissolution in open and closed carbonate systems (Figs. 7 and 8; Sherlock et al., 1995).

According to Millot et al. (2003), surface runoff is the primary weathering process in areas in northern latitudes, rather than chemical weathering. However, Annaselva sediments indicate chemical weathering through sulfide oxidation and carbonate dissolution (Sherlock et al., 1995), as supported by the variation heatmap (Fig. 7) and XRPD data (Fig. 3). Sulfide oxidation often generates acid mine drainage (AMD), leading to decreased pH and mobilization of toxic elements such as As, Se, B, Pb, Cd, Cu, and Zn (Sherlock et al., 1995; Bidari & Ahgazadeh, 2018; Tabelin et al., 2020). Nonetheless, the underlying carbonate lithology buffers the solution by stabilizing pH, therefore weathering carbonates in the area (Sherlock et al., 1995). This theory is further supported by the lack or low concentration of carbonate minerals in stream sediments, undetectable by XRPD analysis (Fig. 3: Type 1 and Type 2). The stream sediments were sampled only in the part of Annaselva catchment that runs over the mineralized part of the Storviknes formation. This area hosts the largest and the most Cu-rich deposits in the entire Kåfjord area (Simonsen, 2021; Hilmo, 2021).

The XRPD data do not align with the statistical findings from the variation matrix for Annaselva and Brakkelva catchments. Although data variation suggests more intensive oxidation of sulfides in Brakkelva, mineralogical analysis (Fig. 3) does not support this. The Brakkelva catchment is underlain by the Kvenvik volcano-sedimentary sequence. Chemical weathering of mafic rocks typically produces silicate minerals (e.g. halloysite, kaolinite) and Fe(III)-oxyhydroxides (e.g. gibbsite, goethite, and hematite) (Goulart et al., 1998; Soubrand-Colin et al., 2005; Asio & Jahn, 2007). During this process, mafic rock-sourced elements like Ni, Co, V, and even Mg and Ca can be mobilized (Soubrand-Colin et al., 2005; Asio & Jahn, 2007). Mentioned mafic rock weathering involves multiple processes such as hydrolysis, carbonation, oxidation, acid dissolution, and ion exchange. Elements like Mg and Ca are readily solubilized through hydrolysis and carbonation reactions, while trace metals like Ni, Co, and V are released during oxidation and dissolution of specific minerals. These processes collectively mobilize elements into aqueous systems, influencing the geochemistry of surrounding environments. However, the presence of easily weathered amphiboles and feldspars (Fig. 3: Types 2, 3, and 4), in the XRPD data suggests that chemical weathering in the area has been limited (Sherlock et al., 1995). Additionally, the formation of Fe(III)-oxyhydroxides could not be confirmed by XRPD data due to their often amorphous nature (Dold, 2003; Zeng et al., 2008). However, these compounds were identified as significant metal carriers through sequential extraction (discussed further in the text) and identified in stream sediments by Hilmo (2021).

The more pronounced chemical weathering in Annaselva stream sediments compared to Brakkelva can be attributed to the higher reactivity of carbonates *versus* Fe-oxyhydroxides. Filgueiras et al. (2002) highlight that mobilizing carbonate-bound metals, i.e. exchangeable fraction, only requires a change in pH, while mobilizing metals bound to Fe-oxyhydroxides, in other words reducible fraction necessitates changes in Eh conditions. In the Kåfjord area, pore water Eh values are relatively stable. Annaselva shows higher Eh (0.216 – 0.258 V) compared to Brakkelva (Eh = 0.177 V) and Møllneselva (Eh = 0.177−0.281 V) (Hilmo, 2021). While pH values are similar, Annaselva generally has slightly lower pH than Brakkelva, with Møllneselva varying more in both Eh and pH (Hilmo, 2021). Under these Eh conditions, almost all elements of interest are in their mobile forms (Geological Survey of Japan, 2005) and can attach to reactive mineral surfaces (Forbes et al., 1974; Svete et al., 2001;  Lacal et al., 2003; Guo et al., 2014). Such reactive mineral surfaces are characteristic for carbonates and Fe(III)-oxyhydroxides, among others (Forbes et al., 1974; Svete et al., 2001;  Lacal et al., 2003; Guo et al., 2014).

**Fig. 10:**
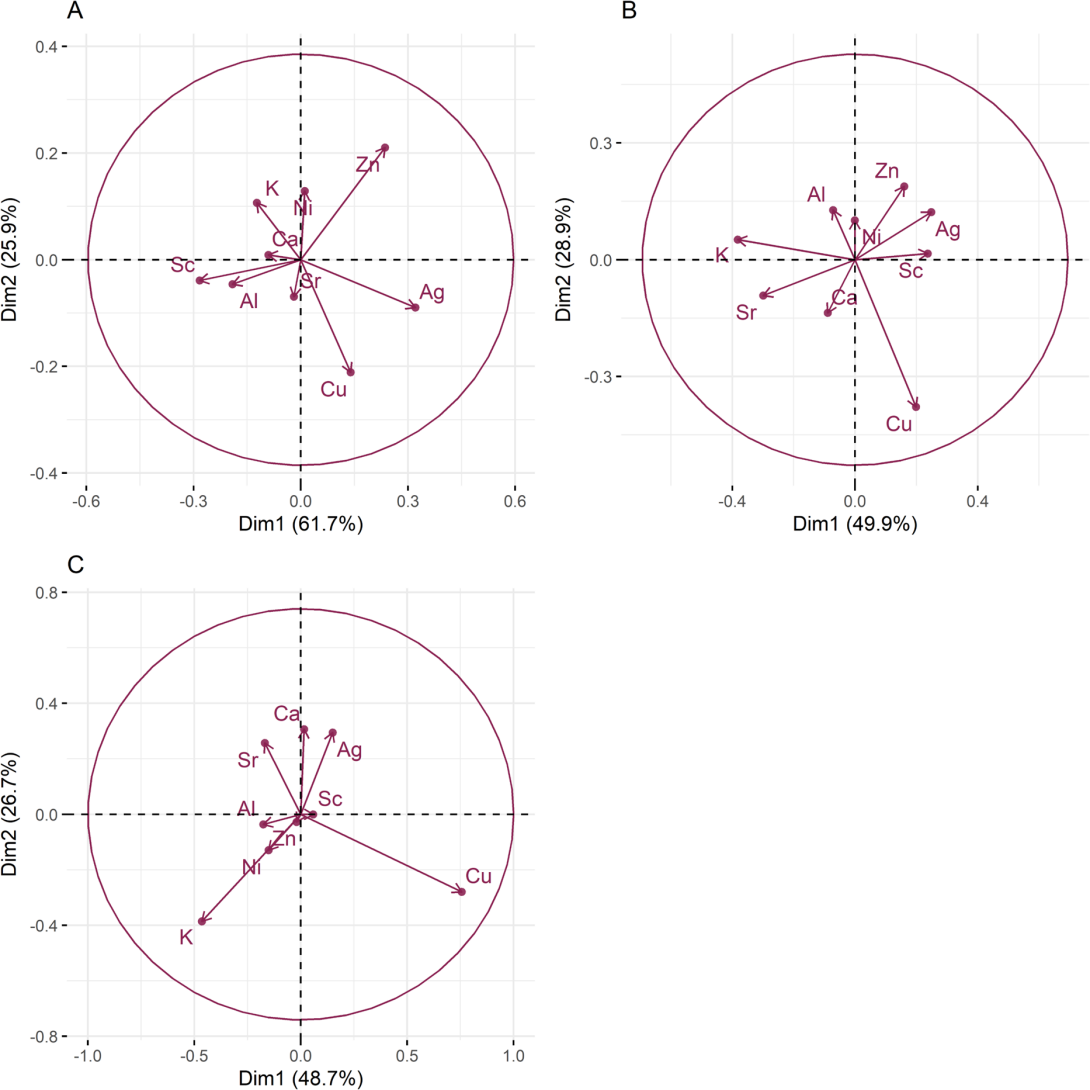
PCA analysis of the bulk geochemical data. In **A** Results for Annaselva are presented with PC1 and PC2 (Dim1 and Dim2) covering 88% of data variability. In **B** Results for Møllneselva are presented, with the first two PCs covering 79% of data variability. In **C** results for Brakkelva are presented with first two PCs covering 75% of data variability

To distinguish occurrence patterns of metals positively correlated with Cu and determine if they are linked to lithology or mineralization, a PCA analysis was conducted. Nine elements were selected for each stream and river. First condition was that elements are representatives for main lithology types present, i.e., Ca and Sr for carbonates, Ni, Sc and Zn for mafic rocks, Al and K for clays. Additionally, Cu and Ag were chosen as representative for the hydrothermal mineralization. The PCA analysis results for sediments from each catchment are shown in Fig. 10.

In PCA analysis of compositional data, the distance between variables can reflect their similarities (van den Boogaart & Tolosana-Delgado, 2013): closer variables show stronger correlations. For Annaselva stream sediments (Fig. 10A) a clear distinction is observed between lithologically derived elements (Al, Ca, K, Ni, Sr, Sc, Zn) and mineralization-associated elements (Cu, Ag). Minerals formed from the erosion and weathering of surrounding rocks such as shales, basalts and Quarternary deposits, form minerals like Qtz, Ill, and Chl, (Figs. 1 and 3), which dominate the stream sediments, grouping lithologically sourced elements. Specifically, Al and K come from clay minerals (Online Resource 8). Ca and Sr as major carbonate cations are uncorrelated in Annaselva (Fig. 10A), indicating that these do not originate from carbonates, but rather are sourced from clay minerals (Wissocq et al., 2017). There is additional division between lithologically derived elements. In Fig. 10A Ni and Zn – mafic sourced elements are grouped together in the same quadrant. Scandium, which is also considered mafic sourced is grouped in the same quadrant as Al and Sr, while all three elements are close to K and Ca in the second quadrant. Such relationship of Sc with clay associated elements could be due to its tendency to enrich both mafic rocks but also bind to clays (Andersen & Elburg, 2025; Zhang et al., 2024).

For Møllneselva (Fig. 10B), the PCA confirms a very low positive correlation between Cu and the other analysed elements. The remaining elements are divided into two groups: Group 1 – Ag, Al, Ni, Sc, and Zn; and Group 2 – Ca, K, and Sr. Most of the Group 1 elements are indicative of mafic lithology, while Group 2 elements suggest carbonates and clay minerals. Notably, Al and K, which should originate from illites identified in the sediments, are placed in different groups, but are in the same quadrant. This suggests their origin from feldspar and/or mica, which were determined by XRPD analysis (Fig. 3). Even though K values align closely with Ca and Sr, they still are plotted in separated quadrant than the rest of the analysed elements (Fig. 10B). Silver is grouped with mafic-rock sourced elements (Ni, Zn, and Sc), which suggests its origin from mafic rocks (Hamaguchi & Kuroda, 1959) rather than hydrothermal mineralization. The distinct groupings of lithologically sourced elements may result from Møllneselva’s perpendicular flow direction relative to the lithological units (Fig. 1), leading to metal mixing along the flow path.

For Brakkelva stream (Fig. 10C), Sc is most closely correlated with Cu, as also indicated by the heatmap (Fig. 9). Potassium is perfectly correlated with Ni and Zn, as they are lying on the same line (van den Boogaart & Tolosana-Delgado, 2013). As geological units in Brakkelva catchment are mostly mafic rocks (basalts), such perfect correlation could indicate mixing of clay minerals with mafic rocks. In support to this claim, a carbonate sourced elements (Ca, and Sr) are plotted close together.

### Sequential extraction analysis

**Fig. 11:**
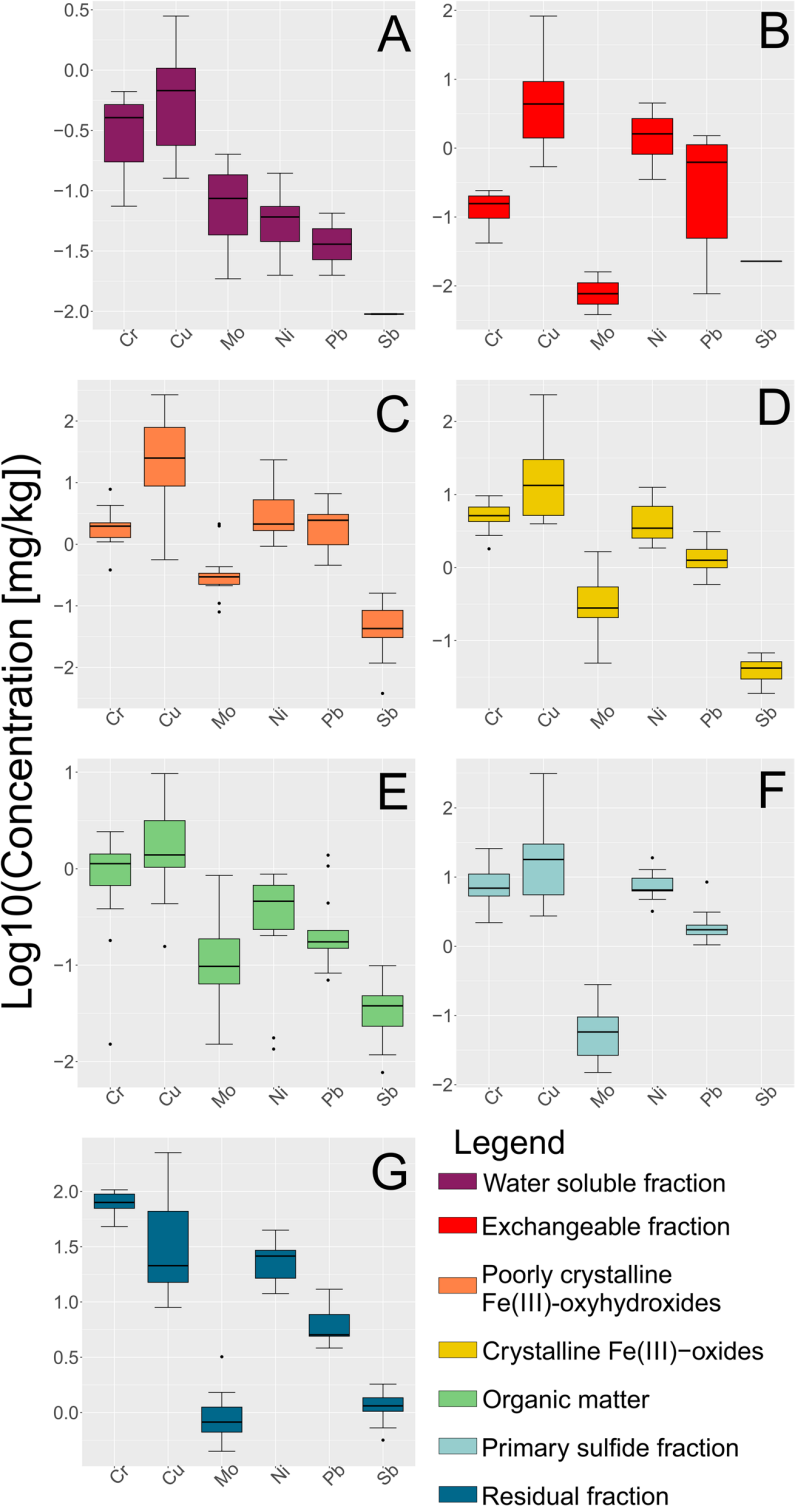
Log10 values of SE measured concentrations. Data was analyzed according to steps, while the colors of the box diagrams correspond to the colors of the fractions in Fig. 12. Black dots represent the suspected outliers. The data represent **A** Water-soluble fraction, **B** Exchangeable fraction, **C** Poorly crystallized Fe(III)-oxyhydroxides, **D** Crystalized Fe(III)-oxides, **E** Organics and Cu-sulfides fraction, **F** Primary sulfides fraction, **G** Residual fraction. The minimum value, 1st quartile, median, 3rd quartile, and maximum values can be seen in the graphs. Visualized using “ggplot2” (RStudio Team, 2022; Wickham, 2016)

The results of the SE analysis are listed in Online Resource 4. Distribution of metals values of the highest interest are visualized in Fig. 11, while all other analysed metals are presented in Online Resource 7.

**Fig. 12:**
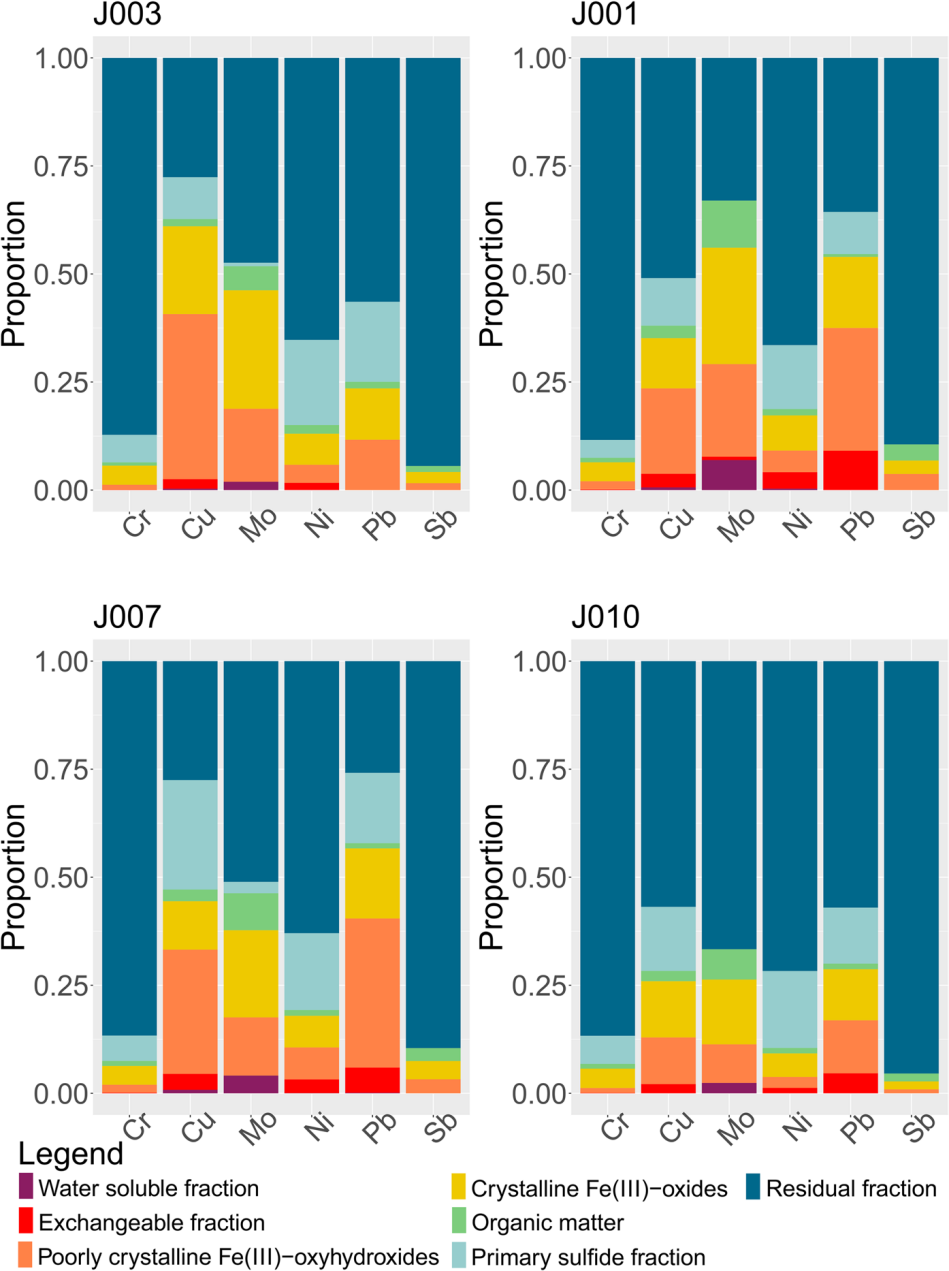
The proportions of metals of interest dissolved from different phases (written in the legend), targeted by sequential extraction in the Annaselva stream

**Fig. 13:**
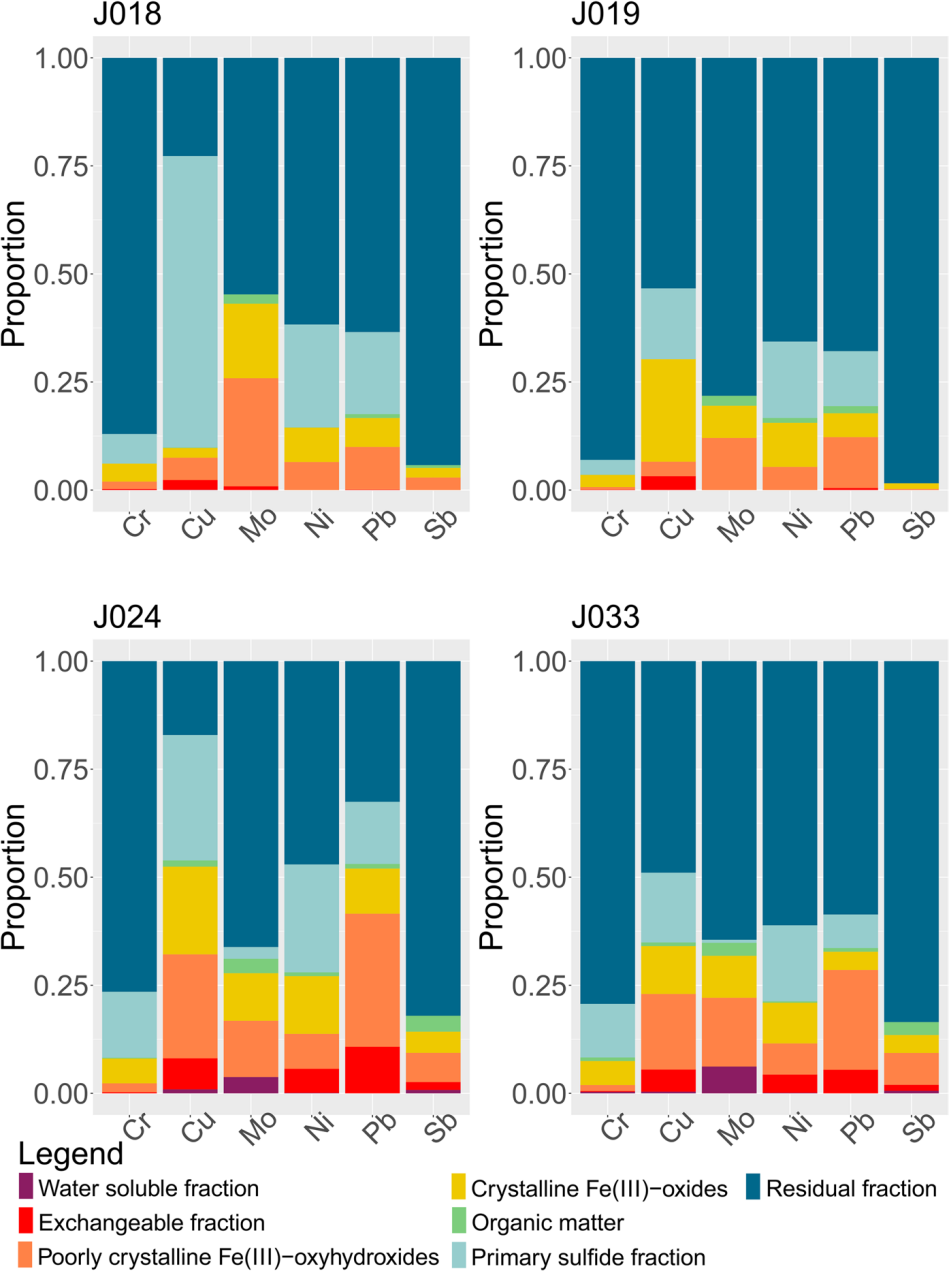
The proportions of metals of interest dissolved from different phases (written in the legend), targeted by sequential extraction in the Møllneselva River

**Fig. 14:**
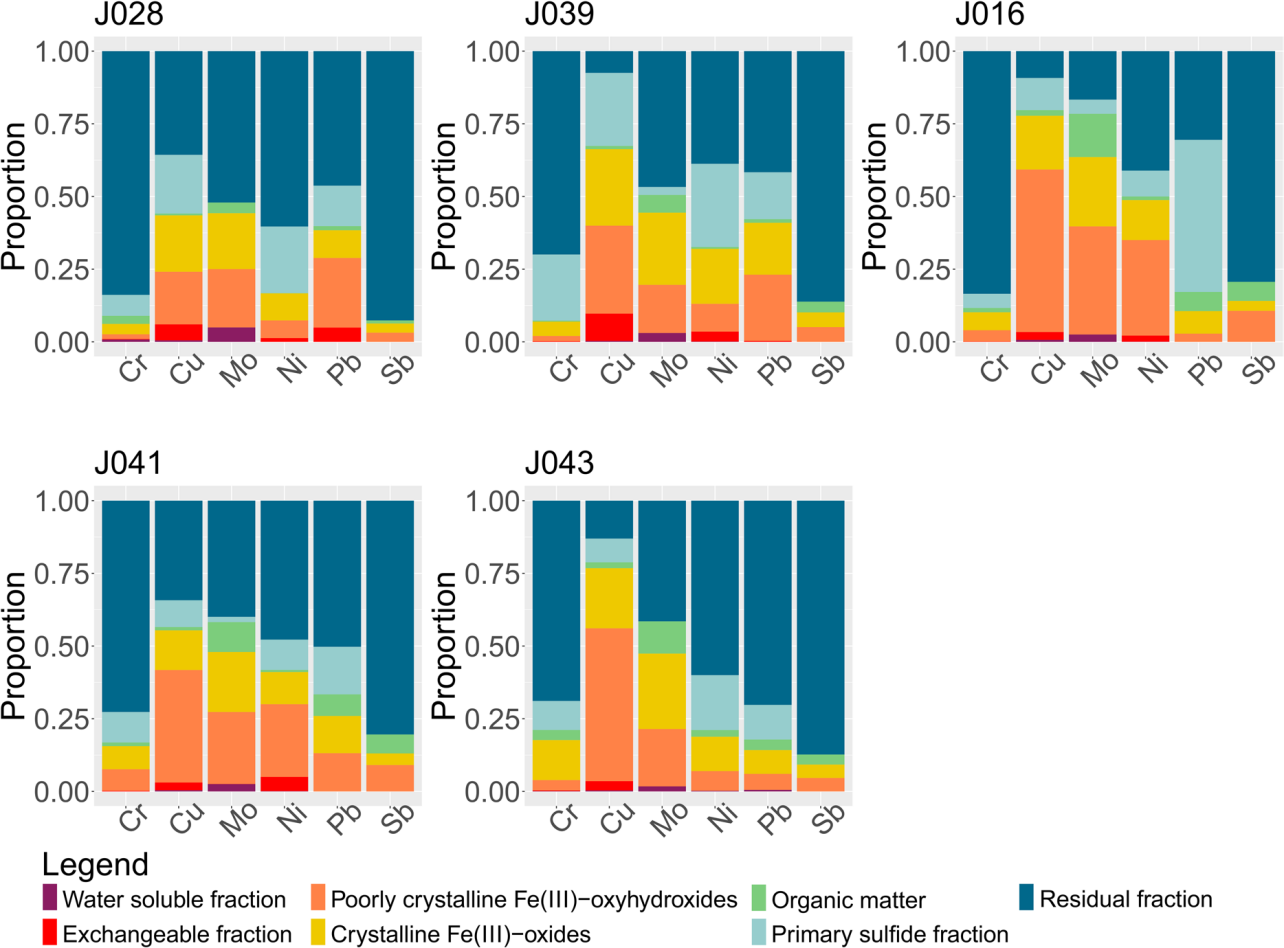
The proportions of metals of interest dissolved from different phases (written in the legend), targeted by sequential extraction in the Brakkelva stream

The results for each stream are presented as stacked diagrams (Figs. 12, 13, and 14). The y-axis shows percentages, i.e., concentrations of metals and metalloids measured for each step of the sequential extraction were transformed into their representative proportions. The sequential extraction of sample J016 was carried out in triplicates (Online Resource 4) - J016a, J016b, and J016c. For the purpose of inspecting trends, the concentrations of elements of interest for this sample were presented as an average value for each step and each metal. The same was done for samples J010 and J041, which were analysed in duplicates, and for sample J033, which was analysed in quadruplets in the seventh step. These multiple measurements and extractions gave information about quality of the procedure.

In all the samples analyzed, the water-soluble fraction is found to be the least prevalent, whereas the residual fraction constitutes the most abundant portion (Figs. 11, 12, 13 and 14 and Online Resources 8 – 10). The water-soluble fraction in all three streams is characterized by detectable concentrations of As, Ba, Cu, Fe, K, Mg, Mn, Mo, Ni, Pb, Rb, Se, Sn, Sr, Ti, U, V, and Y, with the range of concentrations from ≈10^–2^ to ≈10^2^ mg/kg. In almost all samples Be, Ca, Cd, Cr, Li, Sb, and Sc were under their respective detection limits. With the average value of 38.1 mg/kg, Mg shows the highest concentration in all samples (Online Resource 4). Observing all other cumulative concentrations of elements in this fraction, apart from Mg, the highest concentrations, were recorded in the Annaselva sample J001 (33.3 mg/kg) and in Brakkelva sample J041 (27.1 mg/kg). According to Filgueiras et al. (2002), water soluble fraction is small in proportion, but in addition to exchangeable fraction, metals bound onto it are the most mobile and potentially most available to plants.

The concentrations of metals in the exchangeable fraction vary from ≈10^–3^ to ≈10^3^ mg/kg (Fig. 11, and Online Resources 4 and 7). In almost all samples, Li and Sb were under or close to the detection limit. The highest values in this fraction are measured for Ca, as expected, as it is main carbonate cation, followed by Mg and Mn. According to Filgueiras et al. (2002), coprecipitation with carbonates represents important part of the exchangeable fraction. Metals bound onto this fraction are weakly adsorbed and retained on solid surfaces by weak electrostatic interactions, making them easily mobilised (Filgueiras et al., 2002). Barium, Cd, Sr, and U are important minor elements in the exchangeable fraction as well. The proportions of these metals bound onto exchangeable fraction often reach above 15 wt % of the total measured concentrations. In this fraction, the highest values are shown by samples J016 and J041, with the total concentration of all elements of 9339.3 mg/kg (J041) and 11,035.5 mg/kg (J016). Despite carbonate-bound metals being expected in higher quantities in the Annaselva catchment due to its location on the Storviknes sediment sequence, the oxidation of sulfides coupled with carbonate buffering (Lindsay et al., 2015) may have influenced the dissolution of carbonates in Annaselva, showing lower concentrations of metals bound onto exchangeable fraction, as already explained in previous section (“Chemical composition”). The crystalline Fe(III)-oxides fraction is less abundant for the Annaselva compared to Møllneselva and Brakkelva (Fig. 12). Such relationship is expected due to mafic rocks in the base of the stream sediments of Møllneselva and Brakkelva, which weathering forms Fe(III)-oxyhydroxides and Fe(III)-oxides among other weathering products (Goulart et al., 1998; Soubrand-Colin et al., 2005; Asio & Jahn, 2007). The Annaselva stream sediments concentrations in poorly crystalline Fe(III)-oxyhydroxides and crystalline Fe(III)-oxides fractions vary between ≈10^–2^ and ≈10^4^ mg/kg (Fig. 11, and Online Resources 4 and 7). Aluminium, Fe, Mg, and Mn show the highest values (Online Resource 7). In the crystalline Fe(III)-oxides fraction, the maximum Fe values are higher than in the poorly crystalline Fe(III)-oxyhydroxides, for Mn and Ca are lower, and the higher values of Al vary between poorly crystalline Fe(III)-oxyhydroxides and crystalline Fe(III)-oxides. In contrast, Mg shows higher values in the crystalline Fe(III)-oxides fraction compared to the poorly crystalline Fe(III)-oxyhydroxides fraction (Fig. 11, and Online Resource 4). Samples J016 and J041 exhibit the greatest total concentrations for both fractions, surpassing those of the other sediments by an order of magnitude (Online Resource 4). The poorly crystalline Fe(III)-oxyhydroxide and crystalline Fe(III)-oxide fractions are also enriched in As, Cu, Mo, and Pb (Figs. 11, 12, 13 and 14, and Online Resources 8 – 10) in all streams.

In all three streams, the content of metals bound onto the organic fraction is mostly lower than in the all-other analysed fractions, except for water-soluble fraction (Online Resource 4), with an average concentration of around 2.5% of all measured concentrations. The organic fraction has been identified as a main carrier for Se, with 3.63 – 45.6% of the total Se in the SE extracts bound to this fraction in all three streams (Online Resources 4 and 8 – 10). Aluminium (average 122.5 mg/kg), Ca (average 191.4 mg/kg), and Fe (average 117.9 mg/kg) are the elements with the highest measured concentrations in this fraction. Still, these concentrations represent small proportions of their total concentrations extracted in all other fractions combined. Their quantities surpass those of other elements by an order of magnitude. The average concentrations of Be, Cd, Co, Li, Mo, Ni, Rb, Sb, Se, Sn, Sr, U, and V are below 1 mg/kg (Online Resource 4). The Møllneselva River records the lowest quantity of elements bound to this fraction. Samples J016, J041, and J043, all originating from the Brakkelva stream, display the highest cumulative concentrations of elements (Online Resource 4).

The samples J016 and J041 exhibit the highest concentrations across all analysed fractions. This may be attributed to their geographical positions: J016 is located at the inlet of a small creek, while both samples are situated downstream from a bog area. Additionally, the third highest concentrations are characteristic for sample J043, which is positioned downstream from a waterfall and a steeper stream section, suggesting reduced flow velocity (Hilmo, 2021).

The primary sulfide fraction displays element concentrations ranging roughly from ≈10^–2^ to ≈10^4^ mg/kg (Fig. 11, and Online Resources 4 and 7). The most prevalent elements within this fraction are Al, Ca, Fe, and Mg, exhibiting concentrations one to two orders of magnitude higher than the rest, while K is noted for its moderately high concentration (Online Resource 4). Zinc and Cu represent the two most abundant chalcophile elements, with averages in all samples of 13.03 mg/kg and 56.0 mg/kg, respectively (Online Resource 4). All other measured chalcophile elements show concentrations below 1 mg/kg. The samples from the Annaselva stream exhibit the lowest cumulative concentrations of elements, with an average value of 7832.41 mg/kg, in comparison to Møllneselva (12,840.3 mg/kg) and Brakkelva (17,213.41 mg/kg).

To effectively mobilize elements bound to the primary sulfide fraction, a very strong extractant is needed. This extractant induces highly acidic and oxidative conditions (Torres & Auleda, 2013). Among the crucial metals associated with this fraction are Co, Cu, Fe, and Ni (Figs. 12, 13, 14, and Online Resources 4 and 7 – 9). Elevated concentrations of these metals in the primary sulfide fraction are associated with the Møllneselva and Brakkelva (Online Resource 4), indicating bedrock geology, i.e. Co, Fe, and Ni come from mafic rocks; (Fig. 1B) and also from Cu-sulfide mineralization (Cu).

**Fig. 15:**
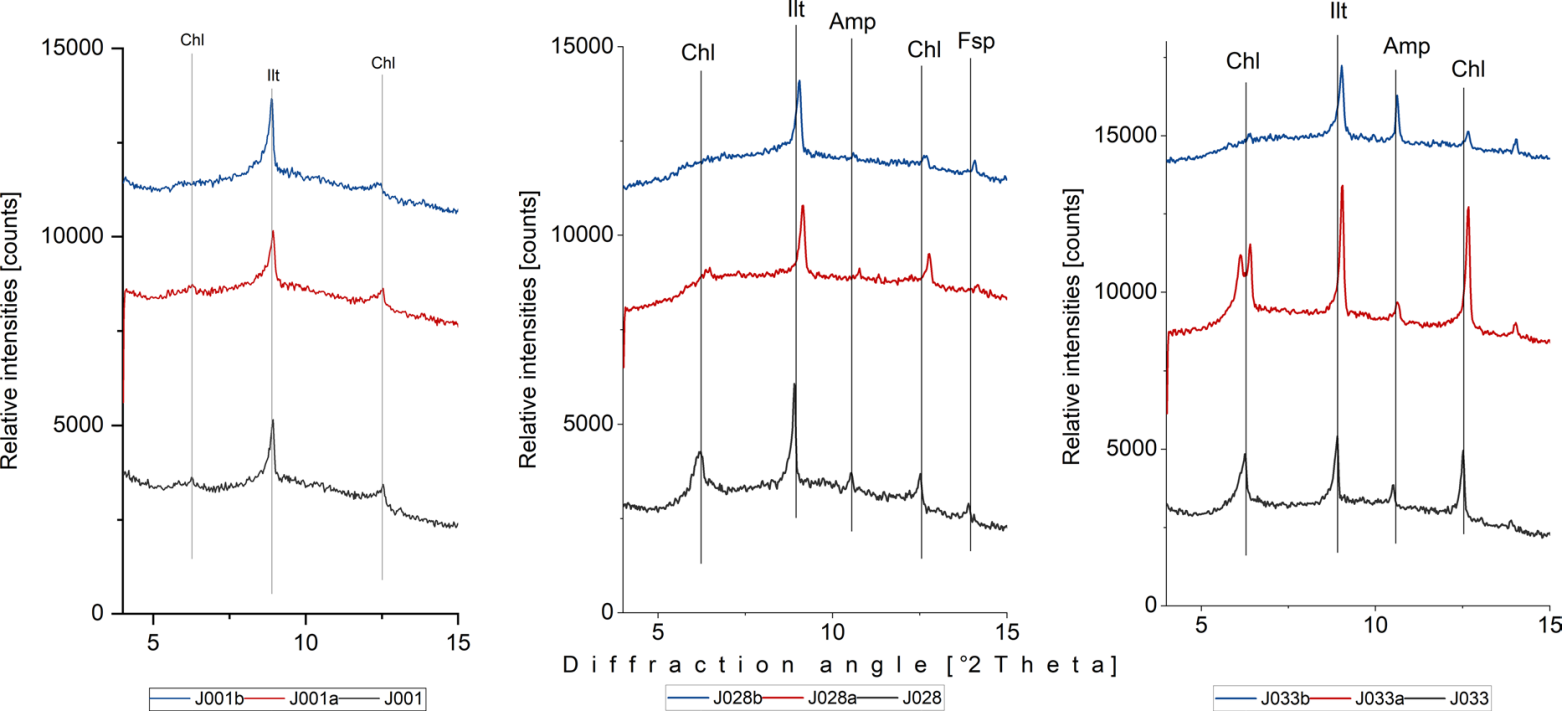
X-Ray diffraction patterns of three samples taken before any treatment (black line), after the 5th step of the sequential extraction (marked with letter a, red line), and after the 6th step of sequential extraction (marked with letter b, blue line). Abbreviations: Chl – chlorite, Ilt – illite, Amp – amphibol, Fsp – feldspar (mineral abbreviations are after Whitney & Evans, 2010)

There is another challenge when analysing SE data, elevated concentrations of lithophile elements such as Ca, K, Li, Mg, and Rb bound to the primary sulfide fraction (Online Resources 8 – 10). These metals cannot be found in sulfide minerals, which was also proved by both Hilmo (2021) and Simonsen (2021) using laser ablation inductively coupled plasma (LA–ICP–MS) on the separated sulfide grains, indicating potential problem in methodology or contamination of the samples. After careful examination of the literature, it is concluded that extracted lithophile elements suggest that the chosen extractant not only targets the primary sulfide fraction, but also affects the crystal lattice of silicate minerals, particularly clays like illite (Liu et al., 2022). Therefore, three representative samples from each stream underwent additional XRPD analysis before the SE procedure, after the 5th step of SE (organic fraction), and once again after the 6th step of SE (primary sulfide fraction). The findings reveal a significant decrease in chlorite maxima intensities after the 6th step (Fig. 15). Conversely, the intensities of illite and quartz maxima remained unchanged. Consequently, we can infer that the partial destruction of the chlorite crystal lattice in reaction with 8 M HNO_3_ led to the mobilization of Ca, K, Li, Mg, and Rb (Snäll & Liljefors, 2000; Kameda et al., 2009).

The residual fraction exhibits the highest proportions of nearly all measured elements per sample (Figs. 11, 12, 13 and 14, and Online Resources 3 and 6 – 9). The distribution of metals bound to the residual fraction generally ranges from 24.54% (Mn) to 95.52% (Ti) of their respective total values when considering all three streams collectively. Aluminium is the most abundant element (average 35,563.89 mg/kg) followed by Fe (average 24,919 mg/kg), K (average 16,188.39 mg/kg), and Mg (average 7045.49 mg/kg). Further details can be found in Online Resource 3.

Despite the challenges associated with extracting the primary sulfide fraction, these findings lead to the conclusion that the residual fraction remains the most abundant fraction for most analysed elements. However, certain lithophile elements are leached out into different fractions, particularly the primary sulfide fraction, which can lead to misinterpretation of the results. Similar challenges were found in studies such as by Dold & Fontboté (2002) and Dold (2003), where a combination of solvents (KClO_3_, HCl, and HNO_3_) was used to treat primary sulfide fraction, resulting in difficulties with the dissolution of certain silicate minerals as well. Nevertheless, aside from elements bound to silicate minerals and challenges associated with primary sulfide fraction, the seven-step SE analysis provides valuable insights into the distribution of elements, especially those associated with Cu-sulfide mineralization, and their potential for mobilization.

### Comparison of statistical and analytical approach

For purposes of this research, several analytical techniques were carried out on the stream and riverine sediments. Of those techniques, semi-total geochemical analysis data provided higher number of samples that produced valuable results after multivariate statistical analyses were carried out. The results of those multivariate analyses were thoroughly examined and compared to the results of all other analytical techniques indicating discrepancies between observed trends.

Statistical variation of data indicates more intensive oxidation of sulfides observed in Brakkelva compared to Annaselva and Møllneselva. This is supported by the lack of significant positive correlation of S to chalcophile elements (Fig. 9). Still, XRPD analysis shows the presence of easily weathered phases, such as feldspars and amphiboles, in Brakkelva sediments, indicating that some of the sulfides should be found. Sequential extraction analysis further supports this assumption with significant concentrations of metals of interest bound to primary sulfide fraction (Fig. 14 and Online Resource 3).

The variation heatmap of the semi-total geochemical analysis data (Fig. 7) shows positive correlation of Cu and Ca (*r* = *0.14*), as well as Cu and Sr (r = 0.06) in Annaselva. These are both considered carbonate-sourced elements, and Cu binding to carbonates could explain these correlations. This relationship, however, is not evident in XRPD data as carbonates were not identified in Annaselva sediments, and stack plots (Fig. 12) highlight that the exchangeable fraction, which usually also involves carbonates, is less significant in Annaselva sediments compared to the fraction of poorly crystalline Fe(III)-oxyhydroxides. This is further supported by PCA analysis, where Ca and Sr are uncorrelated, but positively correlated to clay sourced elements, indicating their binding to illite (Wissocq et al., 2017). However, the variation heatmap partially captures this relationship between Fe(III)-oxyhydroxides and Cu. The variation coefficient between Cu and Fe is *r* = 0.27, whereas the relationship between Cu and Mn (a key carrier in Mn-oxyhydroxides, the most easily reducible fraction according to Filgueiras et al. (2002), which could also be targeted in the 3rd step of sequential extraction), is characterized by a higher coefficient (*r* = 0.89), indicating a weaker correlation. Still, the importance of Fe(III)-oxyhydroxides is shown by Ca binding onto Fe(III)-oxyhydroxides as determined by scanning electron microscopy and energy dispersive X-ray spectroscopy (SEM–EDS) in Hilmo (2021), while correlation between Ca and Cu could be explained by binding of Ca onto Cu-oxides (Simonsen, 2021). The Ca-Fe relationship in Annaselva is also captured by statistical analysis (Fig. 7), where a *log-ratio* between Ca and Fe is *r* = 0.15.

The weathering products of mafic rocks, including Fe(III)-oxyhydroxides, were previously discussed. While literature (e.g. Goulart et al., 1998; Asio & Jahn, 2007) indicates Fe(III)-oxyhydroxides as significant weathering products, XRPD analysis does not confirm their presence due to their amorphous structures. Still, Hilmo (2021) separated them in stream sediments in all three streams and confirmed using SEM–EDS. Additionally, SE analysis reveals that Fe(III)-oxyhydroxides are a key fraction for elements such as Cu, Mo, and Ni in Brakkelva sediments (Fig. 14). The variation matrix does not show significant correlations between Cu and Fe (*r* = 1.02), or Cu and Mn (*r* = 1.72). The correlations for Mo and Ni with Fe and Mn are somewhat better (Fig. 9). Additionally, PCA of Møllneselva stream sediments suggests that Ag correlates better with mafic sourced metals and thus originates from mafic rocks (Hamaguchi & Kuroda, 1959) rather than Cu-sulfide mineralization, but this could not be confirmed by SE analysis since Ag was not analysed.

Zinc and Cu are the primary chalcophile elements associated with the primary sulfide fraction, with concentrations reaching up to 310 mg/kg for Cu and 44 mg/kg for Zn. However, variation matrices show weak correlations between Cu and S and between Zn and S, with higher coefficients indicating lower correlations (Figs. 7, 8 and 9, and Online Resource 3). This indicates yet another discrepancy between statistical approaches on the semi-total geochemical analysis data to trends observed from SE.

These discrepancies between statistical and analytical approaches may arise from the limited sample size and the large number of analysed elements, which can lead to erroneous interpretations (Zhang et al. 2005; Morgan, 2017). The less pronounced relationships between S, Cu, and other elements could also result from differences in geochemical analysis methods (e.g., using *aqua regia* for semi-total geochemical analysis data *versus* stronger reagents for SE analysis), and different extractant to sample mass ratios between methods, which may lead to different extracted and consequently measured concentrations. Despite these issues, the results from both analytical and statistical methods help understand and explain metal mobility and distribution. It is necessary to ensure that statistical analyses are conducted and interpreted with utmost care, considering the limitations of small datasets.

## Conclusions

The Alta-Kvænangen Tectonic Window is an ideal location for studying the geochemical halos of sediment-hosted Cu deposits. Within this region, the Annaselva catchment is notable for its carbonate-buffered system, which reduces the potential for AMD. In contrast, the Brakkelva catchment, characterized by prevailing mafic lithologies, has a weaker buffering capacity.

Through various methodological approaches and analytical techniques, we aimed to identify metals associated with deposits and assess their spatial distribution and binding capacities. Mineralogically, the samples were categorized into four types, with Annaselva sediments falling into only two types (Type 1 – quartz and clay minerals and Type 2 – clay minerals, quartz and amphiboles), while sediments from Møllneselva and Brakkelva were classified into all four types.

The spatial analysis of semi-total bulk geochemistry revealed that some of the analysed elements are concentrated near mine openings, tailings disposal sites, after dams where water flow energy is reduced, and at the inlets of creeks into larger streams. Statistical analysis of the same dataset included variation of the data in form of variation heatmap and PCA for correlation of the several most representative metals. Variation heatmaps identified a few elements with significant positive correlations to Cu: in Annaselva, chalcophile elements Ag, Sb, Bi, Pb, Zn, Ni, Tl, Hg, and lithophile elements Sr, Ba, Ca, Al, K; in Møllneselva, only Sc showed a significant correlation; and in Brakkelva, no significant correlations were found. PCA was effective in distinguishing lithological groups and identified Ag as the best positively correlated metal to Cu in Annaselva and Sc in Brakkelva sediments.

The SE analysis unveiled trends in the binding of elements onto different solid fractions. In the water-soluble fraction, metals are bound in the following order based on their proportion of total measured concentrations: Se > Mo > Zn > As > K. Approximately 40% of all measured Cd is bound onto the exchangeable fraction, followed by Ca, Mn, Ba, U, and Sr, with proportions ranging from 11 to 27%. The reducible fractions, i.e. poorly crystalline Fe(III)-oxyhydroxides and crystalline Fe(III)-oxides, bind almost two-thirds of the total As and detectable amounts of Cd, Co, Cu, Fe, Mn, Mo, and U. Selenium exhibits the most significant association with organic matter. The primary sulfide fraction exhibits enrichment in Co, Cu, Fe, and Ni. However, during this phase of the analysis, some lithophile elements (Al, Ca, Mg, and even K) are released from the clay mineral lattice. The residual fraction represents the most abundant component among all analysed samples.

The statistical approach yielded some different conclusions compared to the trends observed through analytical techniques. Significantly positively correlated elements identified by various methods differed for each stream. Nonetheless, spatial analysis consistently showed higher concentrations of certain elements (Ag, As, Ba, Bi, Cd, Cr, Hg, Mo, Ni, Pb, Sb, Se, and Zn) near mines and downstream. Sequential extraction analysis revealed binding sites for some of these elements, showing that the strong positive correlation between Cu and Ca, being in Annaselva, less pronounced in the SE data, than in the statistical analysis results of the semi-total geochemical analysis data. Conversely, according to SE data the variation coefficients between Cu and Fe should be more pronounced (i.e., lower) across all three systems.

## Supplementary Information

Below is the link to the electronic supplementary material.

Supplementary file1 (XLSX 23 KB)

Supplementary file2 (XLSX 10 KB)

Supplementary file3 (XLSX 36 KB)

Supplementary file4 (XLSX 54 KB)

Supplementary file5 (TIF 12358 KB)

Supplementary file6 (TIFF 10388 KB)

Supplementary file7 (TIF 9737 KB)

Supplementary file8 (TIFF 9394 KB)

Supplementary file9 (TIFF 9128 KB)

Supplementary file10 (TIFF 9085 KB)

## Data Availability

No datasets were generated or analysed during the current study.
